# Fueling the heartbeat: Dynamic regulation of intracellular ATP during excitation–contraction coupling in ventricular myocytes

**DOI:** 10.1073/pnas.2318535121

**Published:** 2024-06-12

**Authors:** Paula Rhana, Collin Matsumoto, Zhihui Fong, Alexandre D. Costa, Silvia G. Del Villar, Rose E. Dixon, L. Fernando Santana

**Affiliations:** ^a^Department of Physiology and Membrane Biology, School of Medicine, University of California, Davis, CA 95616

**Keywords:** calcium, mitochondria, mitofusin 2, electrometabolic coupling

## Abstract

The heart beats approximately 100,000 times per day in humans, imposing substantial energetic demands on cardiac muscle. Adenosine triphosphate (ATP) is an essential energy source for normal function of cardiac muscle during each beat, as it powers ion transport, intracellular Ca^2+^ handling, and actin–myosin cross-bridge cycling. Despite this, the impact of excitation–contraction coupling on the intracellular ATP concentration ([ATP]_i_) in myocytes is poorly understood. Here, we conducted real-time measurements of [ATP]_i_ in ventricular myocytes using a genetically encoded ATP fluorescent reporter. Our data reveal rapid beat-to-beat variations in [ATP]_i_. Notably, diastolic [ATP]_i_ was <1 mM, which is eightfold to 10-fold lower than previously estimated. Accordingly, ATP-sensitive K^+^ (K_ATP_) channels were active at physiological [ATP]_i_. Cells exhibited two distinct types of ATP fluctuations during an action potential: net increases (Mode 1) or decreases (Mode 2) in [ATP]_i_. Mode 1 [ATP]_i_ increases necessitated Ca^2+^ entry and release from the sarcoplasmic reticulum (SR) and were associated with increases in mitochondrial Ca^2+^. By contrast, decreases in mitochondrial Ca^2+^ accompanied Mode 2 [ATP]_i_ decreases. Down-regulation of the protein mitofusin 2 reduced the magnitude of [ATP]_i_ fluctuations, indicating that SR-mitochondrial coupling plays a crucial role in the dynamic control of ATP levels. Activation of β-adrenergic receptors decreased [ATP]_i_, underscoring the energetic impact of this signaling pathway. Finally, our work suggests that cross-bridge cycling is the largest consumer of ATP in a ventricular myocyte during an action potential. These findings provide insights into the energetic demands of EC coupling and highlight the dynamic nature of ATP concentrations in cardiac muscle.

## Significance

The function of the heart is to pump blood through the systemic and pulmonary circulations. To do this, cardiac muscle requires ATP as an energy source. Our study reveals lower diastolic cytosolic ATP levels than previously estimated, necessitating a reevaluation of cardiac energy models. Real-time measurements showed beat-to-beat ATP fluctuations, emphasizing dynamic energy regulation during excitation–contraction coupling. We identify two ATP fluctuation modes dependent on sarcoplasmic reticulum-mitochondrial coupling strength. This study compels modification of current models of cardiac energetics and provides mechanistic insights into how ATP fluctuations can regulate multiple signaling pathways, excitability, and contraction in health and disease.

Each cardiac cycle starts when an action potential (AP) is produced by pacemaking cells in the sino-atrial node and propagates cell-to-cell via gap junctions to the adjacent atrial myocytes and eventually to the right and left ventricles. The process by which this AP induces cardiac muscle contraction is called excitation–contraction (EC) coupling. During EC coupling, membrane depolarization activates Ca_V_1.2 channel clusters in the sarcolemma of ventricular myocytes, allowing a small amount of Ca^2+^ to enter the cytosolic nanodomain that separates the sarcolemma and junctional sarcoplasmic reticulum (jSR). This produces an increase in the local intracellular Ca^2+^ concentration ([Ca^2+^]_i_) that activates clusters of type 2 ryanodine receptors (RyR2s) in the jSR via a Ca^2+^-induced Ca^2+^-release mechanism (1), producing a Ca^2+^ spark (2). The synchronous activation of multiple Ca^2+^ sparks during the AP throughout the myocyte summate to produce a global rise in [Ca^2+^]_i_ (3–5).

As [Ca^2+^]_i_ increases, Ca^2+^ binds to troponin C, which is part of the tripartite troponin complex consisting of troponin I, T, and C. This binding event induces a conformational change in the troponin complex, causing troponin I to move. As a result, troponin T and tropomyosin are displaced, exposing the myosin-binding sites on the actin filaments. The binding of myosin heads to the exposed actin sites initiates cross-bridge cycling, where ATP hydrolysis plays a vital role. This cycle of ATP hydrolysis and cross-bridge formation continues as long as Ca^2+^ remains bound to troponin C and drives the contraction of the ventricular myocardium to pump blood to the systemic and pulmonary circulations.

The relaxation phase of the cardiac cycle begins with the termination of Ca^2+^ release and subsequent extrusion of Ca^2+^ from the cytosol to restore to diastolic [Ca^2+^]_i_ levels. Two key proteins are primarily responsible for this extrusion: the SR Ca^2+^ ATPase (SERCA2a) and sarcolemmal Na^+^/Ca^2+^ (NCX) exchanger (6, 7). SERCA2a transports Ca^2+^ back into the SR lumen at the expense of ATP while NCX uses the electrochemical gradient set up by the Na^+^/K^+^ ATPase to transport Ca^2+^ out of the cell in exchange for Na^+^. As [Ca^2+^]_i_ decreases, Ca^2+^ dissociates from troponin C, allowing the troponin complex to return to its initial position. This repositioning of the troponin complex prevents the formation of cross-bridges, thereby causing relaxation and thus a drop in pressure that allows the ventricles to refill with blood.

This description of events underlying EC coupling emphasizes the fact that each cardiac beat requires a significant amount of energy in the form of ATP. This energy is necessary to sustain the electrochemical gradients required for the movement of Ca^2+^, Na^+^, and K^+^ ions across the SR and sarcolemma via ion channels, exchangers, and pumps as well as for cross-bridge cycling. These processes are, in turn, regulated by G protein–coupled protein receptor signaling pathways involving molecules such as phosphatidylinositol 4,5-biphosphate (PIP_2_) (e.g., angiotensin II signaling) and cAMP synthesis (e.g., β-adrenergic receptor signaling). PIP2 and cAMP production requires ATP hydrolysis (8, 9). Thus, EC coupling and its regulation by the autonomic nervous system and other physiological processes is energetically costly.

Oxidative metabolism in the mitochondria supplies about 95% of the ATP required for these processes (10). Classic work by Denton et al. (11–13) provides insights into the mechanisms by which Ca^2+^ dynamics in cardiac muscle are linked with ATP production by mitochondria. Three Ca^2+^-sensitive dehydrogenases—pyruvate dehydrogenase, isocitrate dehydrogenase, and 2-oxoglutarate dehydrogenase—are expressed in cardiac mitochondria and reach their full activity at a free mitochondrial Ca^2+^ concentration of ~1 μM. These enzymes are central to the tricarboxylic acid (TCA) cycle that drives ATP synthesis. Tethering of mitochondria to the SR by the protein mitofusin 2 (Mfn2) plays a key role in coupling [Ca^2+^]_i_ elevations to ATP synthesis (14–16).

While each cardiac cycle necessitates energy consumption, the temporal and spatial dynamics of ATP demands and generation in ventricular myocytes are poorly understood. Indeed, the generally accepted view is that ATP levels range from 8 to 10 mM and do not vary under physiological conditions (17, 18). These findings raise an important question: How can intracellular ATP concentration ([ATP]_i_) remain unchanged in the whole heart during each beat given the metabolic demands of cardiac EC coupling?

To address this question, we performed real-time measurements of cytosolic ATP concentrations ([ATP]_i_) in firing ventricular myocytes with high temporal and spatial resolution using a fluorescent ATP reporter (19). Contrary to long-held models, we show that [ATP]_i_ is lower than 1 mM and varies rapidly during an AP. The magnitude of these fluctuations depends on the strength of SR Ca^2+^ release and is coupled to mitochondrial Ca^2+^ uptake. Collectively, our findings establish that ATP is dynamically regulated during EC coupling, a regulatory paradigm with potentially profound significance for electrometabolic coupling control.

## Results

### Action Potential–Induced Fluctuations in [ATP]_i_ During Cardiac EC Coupling.

**Fig. 1.**
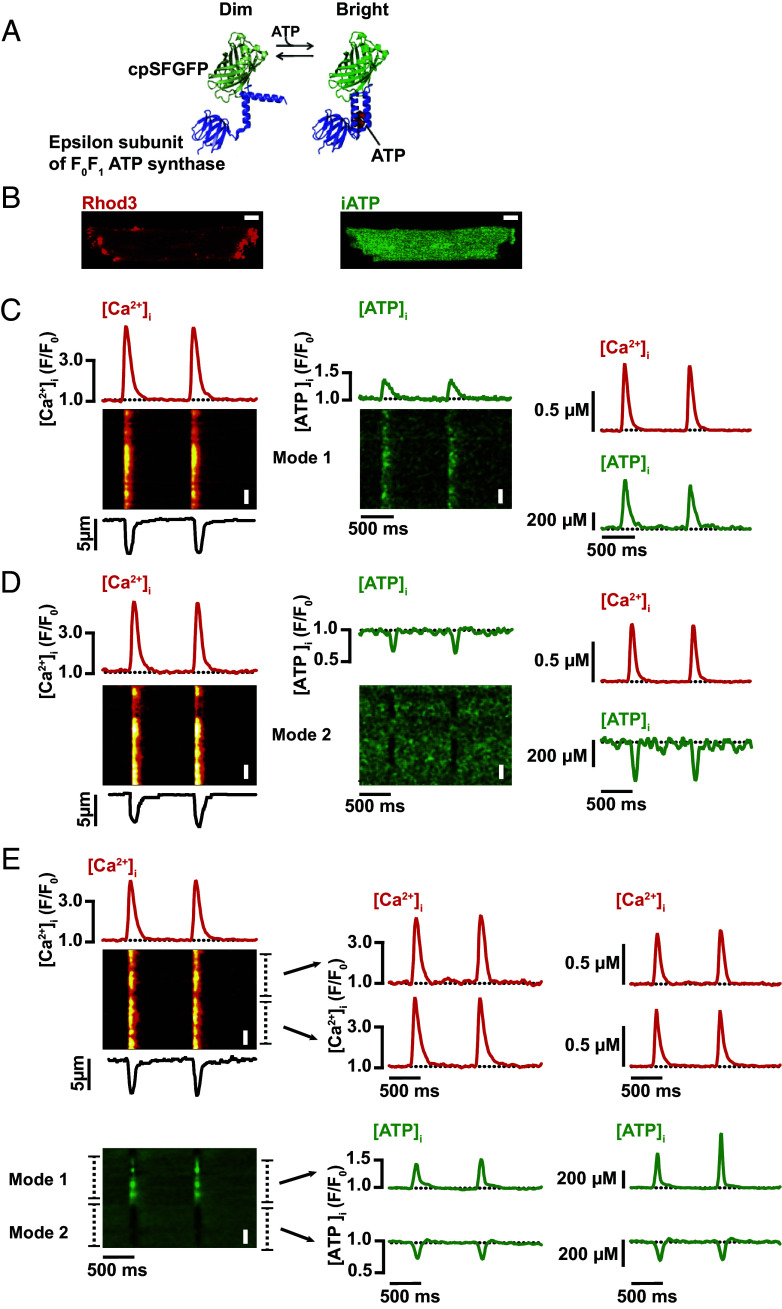
Two different modalities of [ATP]_i_ fluctuations during EC coupling. (*A*) Cartoon of the structure of iATP, which was created by fusing a circularly permuted superfolder GFP (cpSFGFP) with the epsilon subunit of the F_0_F_1_ ATP synthase. iATP increases its fluorescence upon binding ATP. (*B*) Images of a representative ventricular myocyte expressing iATP loaded with the fluorescent Ca^2+^ indicator Rhod-3. Line-scan images of [Ca^2+^]_i_ (*Left* column) and [ATP]_i_ (*Center* column) in a myocyte with cell-wide Mode 1 (*C*) or Mode 2 (*D*) ATP dynamics. The red traces above the [Ca^2+^]_i_ line scans show the cell-wide time course of [Ca^2+^]_i_. The black traces show the time course of cell length. The green traces above the [ATP]_i_ line scans show the cell-wide time course of [ATP]_i_. (*E*) Representative cell with Mode 1 and 2 sites. The time courses for [Ca^2+^]_i_ and [ATP]_i_ in these sites (marked by dashed lines) are shown to the right of each image. The right column shows the same [Ca^2+^]_i_ and [ATP]_i_ traces in the left and center column in μM units. White bars in each image are 10 μm long.

To measure ATP concentration in live ventricular myocytes, we performed retro-orbital injections to infect adult mice with an intracellular ATP sensor, iATP (19) packaged within the cardiotropic adeno-associated virus serotype 9 (AAV9). iATP is a genetically encoded, single-wavelength, cytosolic ATP sensor consisting of a circularly permutated GFP fused to the epsilon subunit of F_0_F_1_-ATP synthase (Fig. 1*A*). This biosensor reports a rise in [ATP]_i_ with an increase in its emitted fluorescence intensity while a decrease in [ATP]_i_ is reported with a decrease in intensity. Importantly, the sensor is insensitive to ADP, AMP, and adenosine. To simultaneously monitor [ATP]_i_, [Ca^2+^]_i_, and contraction, iATP-transduced ventricular myocytes were loaded with the red-shifted Ca^2+^ indicator Rhod-3 AM (Fig. 1*B*) and paced at a rate of 1 Hz.

Confocal line-scan imaging was performed to record [Ca^2+^]_i_ transients and [ATP]_i_ during pacing. Surprisingly, this protocol revealed not just a transient increase in [Ca^2+^]_i_ with each stimulated AP, but an accompanying change in [ATP]_i_. In 57% of myocytes, [ATP]_i_ transiently increased with each evoked AP (Fig. 1*C*). In sharp contrast, 16% of myocytes displayed a decrease in [ATP]_i_ during EC coupling (Fig. 1*D*). A third subpopulation of 27% myocytes displayed a mixed response where some regions along the line scan displayed an increase in [ATP]_i_, while adjacent areas displayed decreased [ATP]_i_ (Fig. 1*E*).

These data indicate that [ATP]_i_ levels vary during an AP and further reveal the existence of three distinct ATP fluctuation modalities in ventricular myocytes. We refer to these modalities as Mode 1, Mode 2, and Mode 1&2. In Mode 1, [ATP]_i_ increases transiently but recovers before the next AP is generated. In Mode 2, [ATP]_i_ decreases transiently in myocytes during the AP but also recovers. While in Mode 1&2, some regions increase, some decrease, and in both cases, a baseline [ATP]_i_ is recovered between beats.

The observation that iATP fluorescence increases during an AP suggests that intracellular ATP levels in ventricular myocytes are close to the reported apparent K_d_ (~120 μM) (19), which would be much lower than the previously estimated 8 to 10 mM level measured with multiple approaches, including NMR (17, 18).

Because the apparent K_d_ of iATP for ATP was not determined in ventricular myocytes, we investigated the relationship between [ATP]_i_ and iATP fluorescence in ventricular myocytes using two complementary approaches (*SI Appendix*, Fig. S1*A*). The first approach involved exposure of β-escin-permeabilized myocytes expressing iATP to a solution designed to mimic the intracellular environment containing ATP concentrations ranging from 100 μM to 10 mM. For the second strategy, we voltage-clamped iATP-expressing myocytes with patch pipettes filled with an intracellular solution containing a similar range of ATP concentrations. As reported by Lobas et al. (19), we detected stepwise increases in iATP fluorescence as ATP increased. Our data suggest that the apparent K_d_ of iATP in ventricular myocytes measured using both approaches was 1,460 μM. The increase in iATP fluorescence from low to saturating levels of ATP was 3.8 ± 0.4-fold.

Having determined these key parameters, we used the maximum fluorescence (F_max_) approach developed by Maravall et al. (20) to convert iATP fluorescence intensity values to μM units in individual cells using their own F_max_ values at 10 mM ATP. Using this approach, we estimated that diastolic [ATP]_i_ was 457 ± 47 μM (*SI Appendix*, Fig. S1*B*).

[Ca^2+^]_i_ transient amplitudes in myocytes exhibiting Mode 1 activity was 2.95 ± 0.17 F/F_0_ (725 ± 26 nM), similar to 3.08 ± 0.33 F/F_0_ (750 ± 44 nM) in myocytes exhibiting Mode 2 activity. In myocytes with Mode 1&2 mixed activity profile, we restricted our analysis to “local” sites, where we measured an [Ca^2+^]_i_ transient amplitude of 2.86 ± 0.11 F/F_0_ (683 ± 14 nM) at regions coincident with the changes in [ATP]_i_ at local Mode 1 sites and 2.92 ± 0.19 F/F_0_ (711 ± 30 nM) at local Mode 2 sites (*SI Appendix*, Fig. S2*A*).

The amplitude of the global transient increase in [ATP]_i_ in Mode 1 cells was 1.22 ± 0.02 F/F_0_ (601 ± 18 μM). In contrast, the average amplitude of the transient decrease in [ATP]_i_ in myocytes exhibiting only Mode 2 regions was 0.94 ± 0.01 F/F_0_ (419 ± 5 μM) or a decrease of 38 μM from diastolic levels of [ATP]_i_.

In Mode 1&2 myocytes, the analysis was restricted to individual subcellular sites with Mode 1 or Mode 2 [ATP]_i_ transients. The amplitudes of Mode 1 sites in these cells were 1.46 ± 0.04 F/F_0_, equivalent to 796 ± 34 μM. The amplitude of the transient [ATP]_i_ decrease in Mode 2 sites was 0.70 ± 0.02 F/F_0_ (297 ± 12 μM), which translates to a decrease in ATP to 160 μM from diastolic [ATP]_i_ (*SI Appendix*, Fig. S2*B*).

We also measured the amplitude of contraction in the ventricular myocytes sampled in this dataset and found that myocytes exhibited similar degrees of shortening regardless of their modality (Mode 1: 8.38 ± 0.51%; Mode 2: 8.04 ± 0.76%; Mode 1&2: 8.52 ± 0.57%; *SI Appendix*, Fig. S2*C*). Thus, our analyses indicate that these differences in [ATP]_i_ dynamics occurred while the amplitude of [Ca^2+^]_i_ transients and contraction were similar in cells with Mode 1, Mode 2, or Mode 1&2 activity.

A detailed analysis of the kinetics of [ATP]_i_ and [Ca^2+^]_i_ transients as well as the associated contraction is shown in *SI Appendix*, Fig. S3. The time to 90% of the peak of ATP transient at Mode 1 sites was faster (17.21 ± 1.88 ms) than in Mode 2 sites (23.72 ± 1.78 ms; *P* = 0.0232) (*SI Appendix*, Fig. S3 *A* and *D*, *Top*). This was about 7 ms longer in Mode 1 sites and 14 ms longer in Mode 2 sites than the time to 90% peak of the associated [Ca^2+^]_i_ transients (i.e., 10.43 ± 1.14 ms, Mode 1; 11.38 ± 1.66 ms, Mode 2) (*SI Appendix*, Fig. S3 *B* and *E*, *Top*). It is important to note that although the on or off rates of iATP have not been determined, stopped-flow kinetic analysis by Lobas et al. (19) suggests that the kinetics of iATP in response to changes in ATP are likely slower to that of the Rhod-3 in response to changes in [Ca^2+^]_i_. Thus, the differences in the time course of [ATP]_i_ and [Ca^2+^]_i_ transients is at least in part due to differences in the kinetics of the ATP and Ca^2+^ sensors.

Notably, the time to 90% of the contraction in cells with Mode 2 (38.75 ± 2.65 ms) was slower than cells with Mode 1 (33.54 ± 1.47 ms; *P* = 0.0374) (*SI Appendix*, Fig. S3 *C* and *F*, *Top*). The time to peak of contractions in cells with Mode 1 and 2 sites (36.29 ± 1.96 ms) was intermediate between that of cells with Mode 1 or Mode 2 sites. Finally, our analysis suggests that while the kinetics of decay of the [Ca^2+^]_i_ transient relaxation was similar in cells with all [ATP]_i_ signaling modalities, the decay of [ATP]_i_ transient was faster for Mode 1 than Mode 2 transients (*P* = 0.0003) (*SI Appendix*, Fig. S3 *D*, *Bottom*).

The temporal relationships between [Ca^2+^]_i_, [ATP]_i_, and contraction are better appreciated in the Ca^2+^–iATP and cell length–iATP plots in *SI Appendix*, Fig. S4. These plots show that as [Ca^2+^]_i_ increased, iATP remained relatively constant at basal levels initially, but then increased (Mode 1) or decreased (Mode 2) as [Ca^2+^]_i_ continued to rise. As [Ca^2+^]_i_ release was terminated and [Ca^2+^]_i_ decayed, iATP began to return back to diastolic levels. Although the variances in the timing of [Ca^2+^]_i_ and [ATP]_i_ fluctuations during an action potential could be reflective of ATP synthesis or utilization post-AP-evoked [Ca^2+^]_i_ changes, as noted above, the observed hysteresis in [Ca^2+^]_i_-[ATP]_i_ loops may be partly attributed to differences in the kinetics of ATP and Ca^2+^ sensors.

Interestingly, in cells with Mode 1, 2, and 1&2 ATP transient modalities, ATP levels increased or decreased with a time course that more closely matched that of cell length—unlike in the [Ca^2+^]_i_-[ATP]_i_ loops—suggesting that contraction and iATP fluorescence transient may have similar kinetics.

### Mitochondrial Oxidative Phosphorylation Contributes to Diastolic and Systolic ATP Production.

**Fig. 2.**
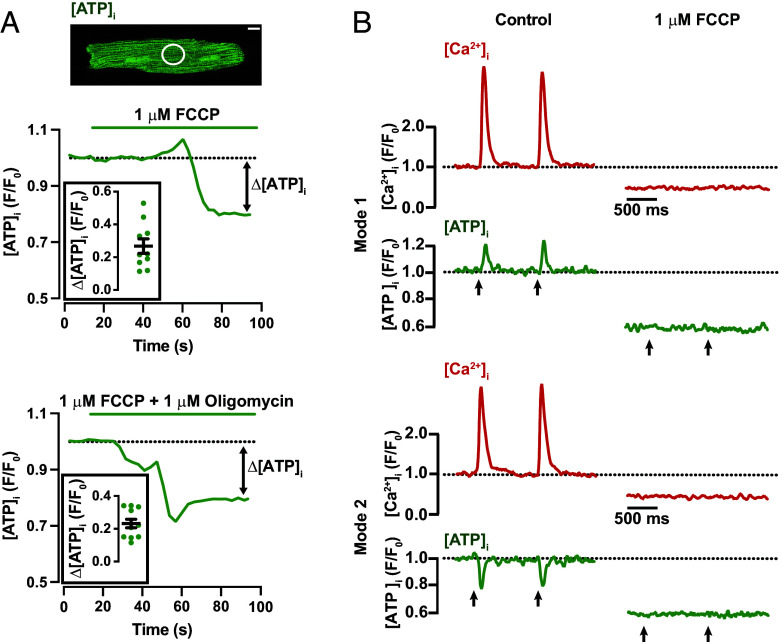
Oxidative phosphorylation is critical for diastolic and [ATP]_i_ transients during contraction. (*A*) Confocal image of an exemplar ventricular myocyte expressing iATP. The plot below the image shows the time course of [ATP]_i_ in the region within the white circle before and after the application of 1 μM FCCP. The plot underneath shows the time course of [ATP]_i_ from a different cell before and after the application of 1 μM FCCP and 1 μM oligomycin. The white bar on the image equals 10 μm. The double-headed arrow indicates the change in [ATP]_i_ in response to inhibition of mitochondrial oxidative phosphorylation, which represents ATP consumption by homeostatic cellular processes. *Insets* show summary change in [ATP]_i_ in response to FCCP (N = 4, n = 10) or FCCP and oligomycin (N = 4, n = 11). (*B*) Line-scan images of [Ca^2+^]_i_ and [ATP]_i_ from 2 ventricular myocytes with Mode 1 and 2 sites before and after FCCP application. The arrows indicate the timing of field stimulation.

We tested the hypothesis that [ATP]_i_ transients were generated by mitochondrial oxidative phosphorylation leading to ATP synthesis, by performing confocal imaging time series on quiescent (i.e., unpaced) ventricular myocytes expressing iATP. The time course of [ATP]_i_ before and after the application of the mitochondrial oxidative phosphorylation uncoupler FCCP (1 μM) alone or FCCP and the ATP synthase inhibitor oligomycin (1 μM) from exemplar myocytes are shown in Fig. 2*A*. Note that exposure to these inhibitors of mitochondrial ATP production led to a decrease in diastolic [ATP]_i_ to a lower steady state (0.27 ± 0.04 F/F_0_ for FCCP, and 0.23 ± 0.03 F/F_0_ for FCCP + oligomycin) within 2 min of exposure to the drug. This Δ[ATP]_i_ suggests that diastolic ATP consumption in a ventricular myocyte is significant.

Next, we recorded [Ca^2+^]_i_ and [ATP]_i_ in control and FCCP-treated ventricular myocytes during pacing (1 Hz) and found that FCCP completely eliminated [Ca^2+^]_i_ and [ATP]_i_ transients (Fig. 2*B*). One potential mechanism by which FCCP may have blocked EC coupling is by decreasing ATP, which would increase ATP-sensitive K^+^ (K_ATP_) currents and hence decrease excitability. Consistent with this hypothesis, we found that application of FCCP followed by FCCP plus the K_ATP_ blocker glibenclamide (10 μM) restored EC coupling in ventricular myocytes (*SI Appendix*, Fig. S5). These data indicate that mitochondria play a crucial role in generating ATP for homeostatic processes during diastole and [ATP]_i_ transients during EC coupling in ventricular myocytes.

### SR Ca^2+^ Release Induces [Ca^2+^]_mito_ Changes at Mode 1 and Mode 2 ATP Fluctuation Sites in Ventricular Myocytes.

**Fig. 3.**
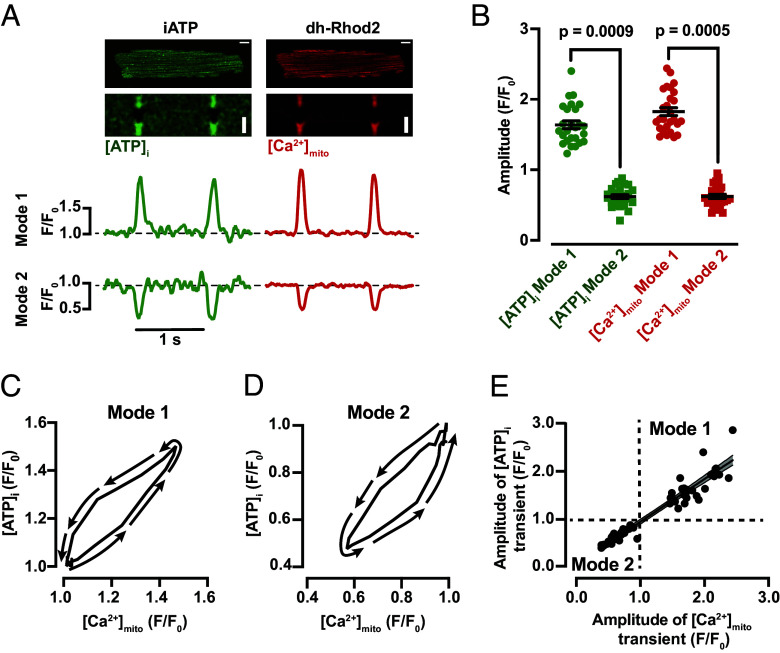
[Ca^2+^]_mito_ changes in Mode 1 and Mode 2 ventricular myocytes. (*A*) Two-dimensional and line-scan confocal images of a representative ventricular myocyte expressing iATP loaded with the mitochondrial fluorescent Ca^2+^ indicator dh-Rhod-2. The traces below each line scan show the time course of [ATP]_i_ and [Ca^2+^]_mito_ in Mode 1 and 2 sites in this cell. White bars in each image are 10 μm long. (*B*) Scatter plot of the amplitudes of [ATP]_i_ and [Ca^2+^]_mito_ transients in Mode 1 and Mode 2 sites (N = 3/n = 27). All significant values are provided from a nested *t* test. The mean values ± SEM of all individual values are in black. Panels (*C*) and (*D*) show the relationship between [ATP]_i_ and [Ca^2+^]_mito_ during the contraction and is plotted as a trajectory. For each trace, the cell begins and ends in a relaxed state at low [ATP]_i_ and [Ca^2+^]_mito_. Thus, the diagrams “begin” and “end” at the intersection of 1.0 in the x- and y- axes. The lines with arrows indicate the trajectory of the relationship. (*E*) Plot showing the relationship between the amplitude of simultaneously recorded [ATP]_i_ and [Ca^2+^]_mito_ signals in individual myocytes. The solid line shows a linear fit to the data with a slope of 0.89 Δ[ATP]_i_/Δ[Ca^2+^]_mito_. The dashed lines show the 95% CI of the fit.

Having determined that mitochondria are necessary for [ATP]_i_ transients during EC coupling, we tested the hypothesis that SR Ca^2+^ release during the AP is coupled to changes in [ATP]_i_. A testable prediction of this hypothesis is that SR Ca^2+^ release induces increases in mitochondrial Ca^2+^ concentration ([Ca^2+^]_mito_) that drive [ATP]_i_ fluctuations. To do this, we recorded [Ca^2+^]_mito_ dynamics in ventricular myocytes expressing iATP loaded with the mitochondrial Ca^2+^ indicator dh-Rhod2 (Fig. 3). Ventricular myocytes were paced at a frequency of 1 Hz.

Before delving into these experiments, we performed two key control experiments. First, we simultaneously imaged mitochondria in living ventricular myocytes expressing iATP and loaded with Mitotracker Far Red and dh-Rhod2 (*SI Appendix*, Fig. S6). Analysis of these images suggested a Pearson correlation coefficient for Mitotracker and dh-Rhod2 was 0.70 ± 0.02, confirming that this Ca^2+^ indicator was localized to the mitochondria (21). Notably, Pearson’s correlation coefficient of Mitotracker and iATP signals of 0.17 ± 0.02 shows that iATP expression is largely cytosolic in ventricular myocytes and that if there is any translocation into the mitochondria, it is minimal. Accordingly, experiments examining subcellular fluorescence recovery after photobleaching (FRAP) of iATP suggested that the sensor is mobile within the cytoplasm with a diffusion coefficient of 1.82 μm^2^ for the fast component and 0.29 μm^2^ for the slow component (*SI Appendix*, Fig. S7).

Having confirmed that iATP and dh-Rhod2 are properly targeted to different cellular compartments, we proceeded to record [Ca^2+^]_mito_ and [ATP]_i_ in beating myocytes. Consistent with the work of Robert et al. (21), we observed beat-to-beat changes in [Ca^2+^]_mito_. Interestingly, we found that in Mode 1 sites, a transient increase in [Ca^2+^]_mito_ was associated with an increase in [ATP]_i_ during the activation of an AP. By contrast, in Mode 2 sites, a transient decrease in [Ca^2+^]_mito_ was associated with a decrease in [ATP]_i_ during the activation of an AP (Fig. 3*A*). The amplitude of [ATP]_i_ was 1.64 ± 0.06 F/F_0_ and 0.62 ± 0.03 F/F_0_ in Mode 1 and 2, respectively while the amplitude of [Ca^2+^]_mito_ was 1.82 ± 0.06 F/F_0_ and 0.62 ± 0.03 F/F_0_ in Mode 1 and 2, respectively (Fig. 3*B*).

We generated [Ca^2+^]_mito_-[ATP]_i_ plots from a representative myocyte displaying Mode 1 (Fig. 3*C*) and Mode 2 (Fig. 3*D*) ATP dynamics. These plots show that the time course of changes in [Ca^2+^]_mito_ closely matched changes in [ATP]_i_ in a Mode 1 and Mode 2 domains. We also plotted the peak of [Ca^2+^]_mito_ and [ATP]_i_ for all cells examined and found that the relationship between these two parameters is linear (Fig. 3*E*). Collectively, our data suggest that increases in [ATP]_i_ are closely associated with increases in [Ca^2+^]_mito_, and vice versa, during the cardiac AP.

**Fig. 4.**
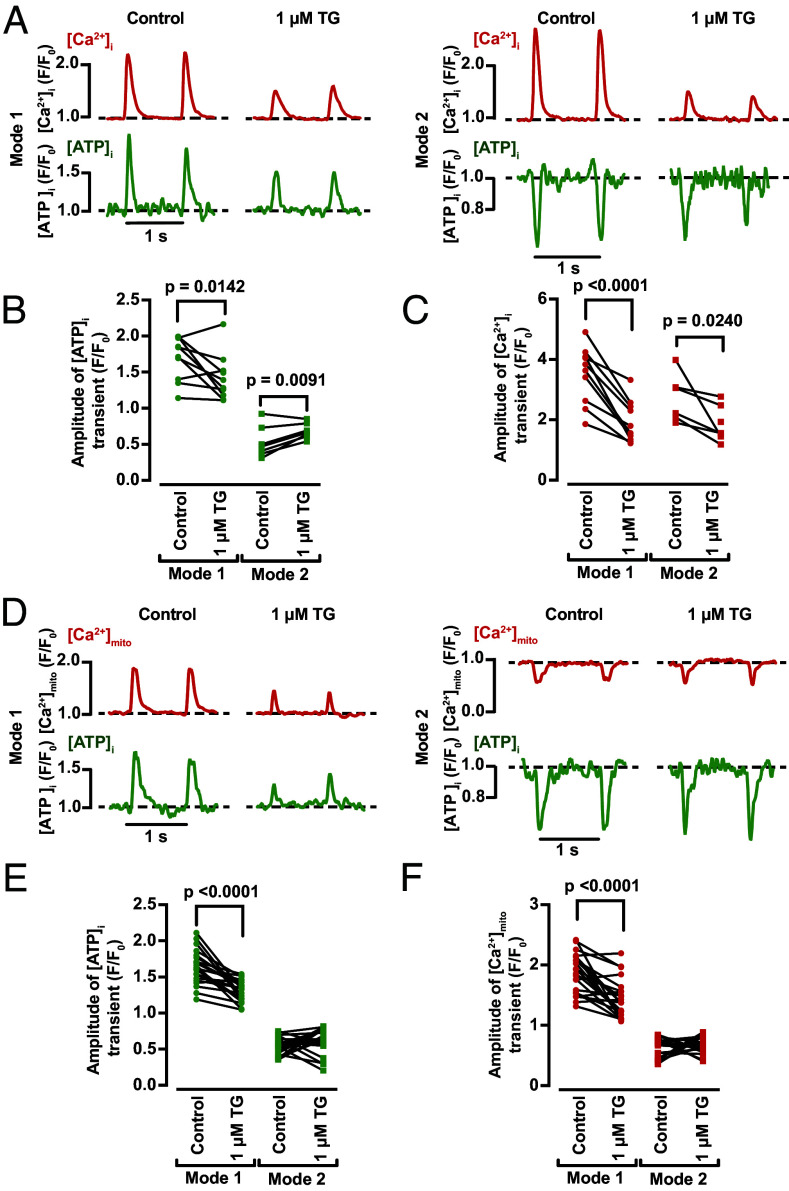
Eliminating SR Ca^2+^ release decreases [Ca^2+^]_mito_ and [ATP]_i_ in ventricular myocytes. (*A*) Time course from line scans of [ATP]_i_ and [Ca^2+^]_i_ in Modes 1 and 2 sites before (*Left*) and after (*Right*) application of 1 μM thapsigargin. Paired scatter plot of the amplitudes of [ATP]_i_ (*B*) and [Ca^2+^]_i_ (*C*) transients in Modes 1 and 2 sites (N = 4, n = 10/7, respectively). (*D*) Time course from line scans of [ATP]_i_ and [Ca^2+^]_mito_ in Mode 1 and 2 sites before (*Left*) and after (*Right*) application of 1 μM thapsigargin. Scatter plot of the amplitudes of [ATP]_i_ (*E*) and [Ca^2+^]_mito_ (*F*) transients in Mode 1 and 2 sites (N = 4, n = 22/20, respectively). All significant values are provided from a paired *t* test.

Next, we tested the hypothesis that changes in cytosolic ATP are driven by SR Ca^2+^ release during EC coupling. To do this, we recorded [ATP]_i_ and [Ca^2+^]_i_ or [Ca^2+^]_mito_ in ventricular myocytes before and after application of the SR Ca^2+^ ATPase inhibitor thapsigargin (1 μM) (Fig. 4). Application of thapsigargin decreased the amplitude of [Ca^2+^]_i_ transients (by 45 and 32% for Mode 1 and Mode 2 myocytes, respectively). Notably, thapsigargin also decreased the amplitude of Mode 1 [ATP]_i_ transients by 16% and Mode 2 transients by 28% (Fig. 4 *B* and *C*). Similarly, reduction of SR Ca^2+^ release with thapsigargin decreased [Ca^2+^]_mito_ and [ATP]_i_ transients in Mode 1 cells by 23% and 19%, respectively (Fig. 4 *D–F*). These data suggest that SR-mitochondrial coupling is important for ATP production during EC coupling. Importantly, because Mode 1 sites in thapsigargin-treated cells still underwent increases in [Ca^2+^]_mito_ and [ATP]_i_ during the AP, these findings suggest that Ca^2+^ influx can also impact ATP dynamics during EC coupling.

### Mitofusin 2 is Important for Mode 1 Ca^2+^–ATP Relationship in Ventricular Myocytes.

One potential mechanism by which SR Ca^2+^ release induces an increase in [Ca^2+^]_mito_ and [ATP]_i_ is via the formation of mitochondrial-SR junctions by mitofusins. Two mitofusins are expressed in the heart: Mitofusin 1 (Mfn1) and 2 (Mfn2) (14, 22). We tested the hypothesis that Mfn2 expression was required for Mode 1 ATP dynamics.

We focused on Mfn2 as it has been proposed to play a key role in tethering mitochondria to the SR and regulating intraorganelle Ca^2+^ cross talk. Furthermore, Ca^2+^-induced stimulation of Krebs cycle dehydrogenases is diminished in Mfn2 null, but not Mfn1 null ventricular myocytes (14). To test our hypothesis, we infected mice with an AAV9 bicistronic vector that expresses an shRNA designed to down-regulate Mfn2 while expressing the iATP sensor only in cells where Mfn2 shRNA was taken up. PCR analysis of hearts from mice infected with this virus suggested that, on average, this strategy resulted in a decrease of about 85% of Mfn2 mRNA transcripts. Importantly, we did not detect any decrease in Mfn1 transcript, underscoring the specificity of our approach (*SI Appendix*, Fig. S8*A*).

**Fig. 5.**
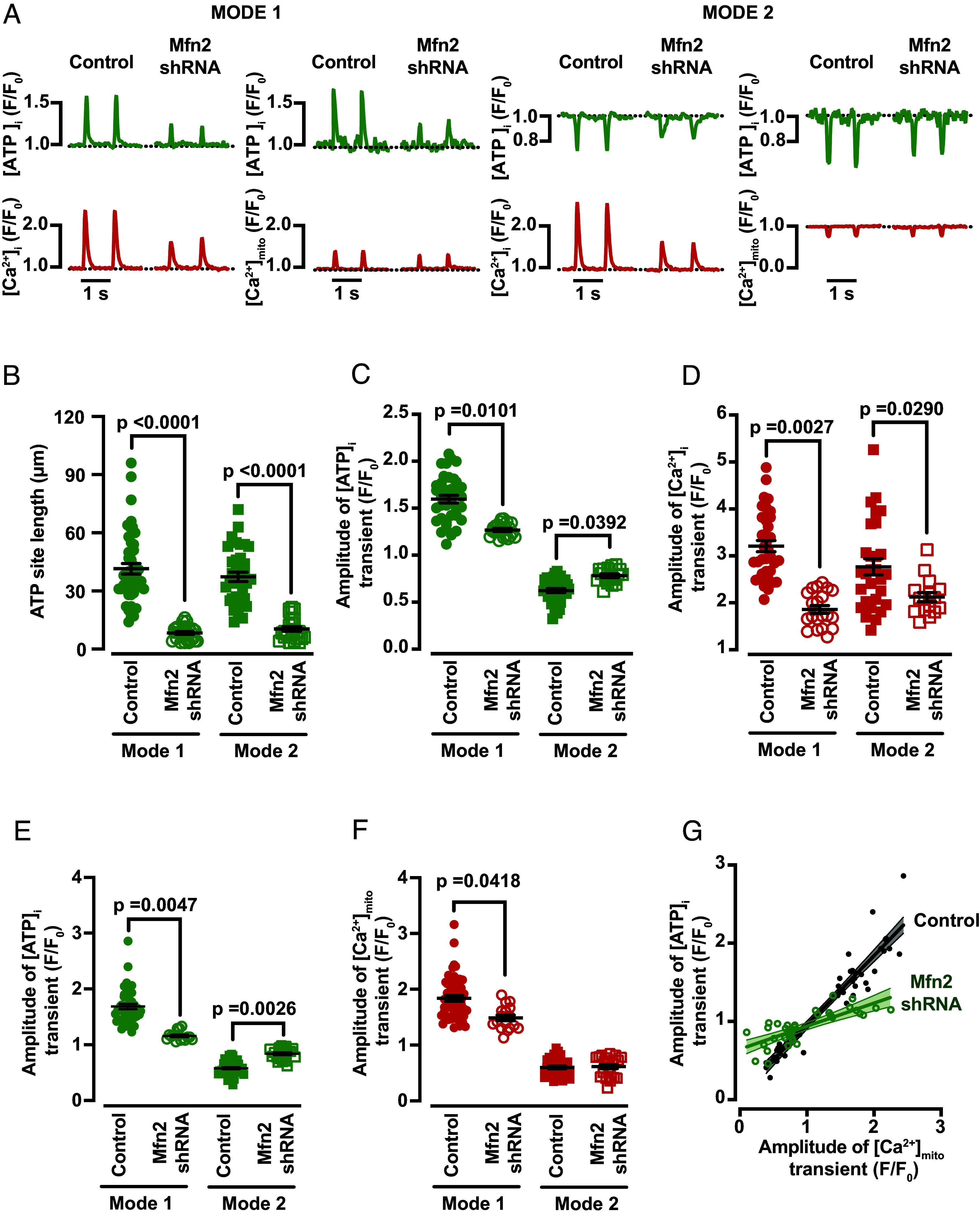
Mitofusin 2 is important for Mode 1 Ca^2+^–ATP relationship in ventricular myocytes. (*A*) Time course from line scans of [Ca^2+^]_i_, [Ca^2+^]_mito_ and [ATP]_i_ in Mode 1 and Mode 2 sites recorded from control and Mfn2-shRNA cells. Scatter plots of the spatial spread of [ATP]_i_ (*B*) (N = 15, n = 45/34 and N = 3, n = 27/24 of Modes 1 and 2 sites in control and Mfn2 shRNA, respectively) and amplitudes of [ATP]_i_ (*C*) and [Ca^2+^]_i_ (*D*) transients in control and Mfn2-shRNA expressing myocytes (N = 8, n = 35 and N = 14, n = 34 of control Mode 1 and Mode 2, and N = 3, n = 23 and N = 3, n = 17 Mfn2 shRNA Mode 1 and Mode 2 sites, respectively). Scatter plots of the amplitudes of [ATP]_i_ (*E*) and [Ca^2+^]_mito_ (*F*) transients in control and Mfn2-shRNA expressing myocytes (N = 4, n = 43/37 of control Mode 1 and Mode 2 sites, and N = 3, n = 15/21 of Mfn2 shRNA Mode 1 and Mode 2 sites, respectively). All significant values are provided from a nested *t* test. The mean values ± SEM of all individual values are in black. (*G*) Plot showing the relationship between the amplitude of simultaneously recorded [ATP]_i_ and [Ca^2+^]_mito_ signals in control cells and cells expressing Mfn2-shRNA. The solid lines show linear fits to the data with a slope of 0.89 Δ[ATP]_i_/Δ[Ca^2+^]_mito_ in control myocytes and 0.29 Δ[ATP]_i_/Δ[Ca^2+^]_mito_ in Mfn2-shRNA myocytes. The dashed lines show the 95% CI of the fits.

Fig. 5*A* shows the time course of [ATP]_i_ and [Ca^2+^]_i_ representative traces from representative ventricular myocytes paced at a frequency of 1 Hz using field stimulation. While Mfn2-deficient myocytes still displayed Mode 1 and 2 [ATP]_i_ transients during an AP, the spatial width (Fig. 5*B*) and amplitude (Fig. 5*C*) of local [ATP]_i_ transients in these sites were significantly smaller than in control myocytes (*P* < 0.0001 for Mode 1 and Mode 2 site length; *P* = 0.0101 and *P* = 0.0392 for Mode 1 and Mode 2 [ATP]_i_ transients’ amplitude, respectively). We found that cells expressing Mfn2-shRNA had smaller [Ca^2+^]_i_ transients than control (Fig. 5*D*). Importantly, differences in [ATP]_i_ were not observed in ventricular myocytes infected with a virus expressing a scrambled shRNA when compared with wild-type iATP-infected myocytes (*SI Appendix*, Fig. S8 *B* and *C*).

Furthermore, we recorded [ATP]_i_ and [Ca^2+^]_mito_ transients in Mfn2-shRNA myocytes (Fig. 5*A*). As expected, [ATP]_i_ amplitude changes were smaller in Mode 1 and 2 sites from Mfn2-shRNA myocytes compared to sites from control myocytes (Fig. 5*E*). We also found that the amplitude of [Ca^2+^]_mito_ transients in Mode 1 sites in Mfn2-deficient myocytes was smaller than in control myocytes (Fig. 5*F*).

Fig. 5*G* shows a plot of the simultaneously recorded peak [Ca^2+^]_mito_ and [ATP]_i_ in control and Mfn2-deficient myocytes. Our analysis suggests that down-regulation of Mfn2 leads to a decrease in the slope of the [Ca^2+^]_mito_-[ATP]_i_ relationship in ventricular myocytes from 0.89 Δ[ATP]_i_/Δ[Ca^2+^]_mito_ in control myocytes to 0.29 Δ[ATP]_i_/Δ[Ca^2+^]_mito_ in Mfn2-shRNA myocytes.

### Contraction Consumes Significant ATP During Cardiac EC Coupling.

**Fig. 6.**
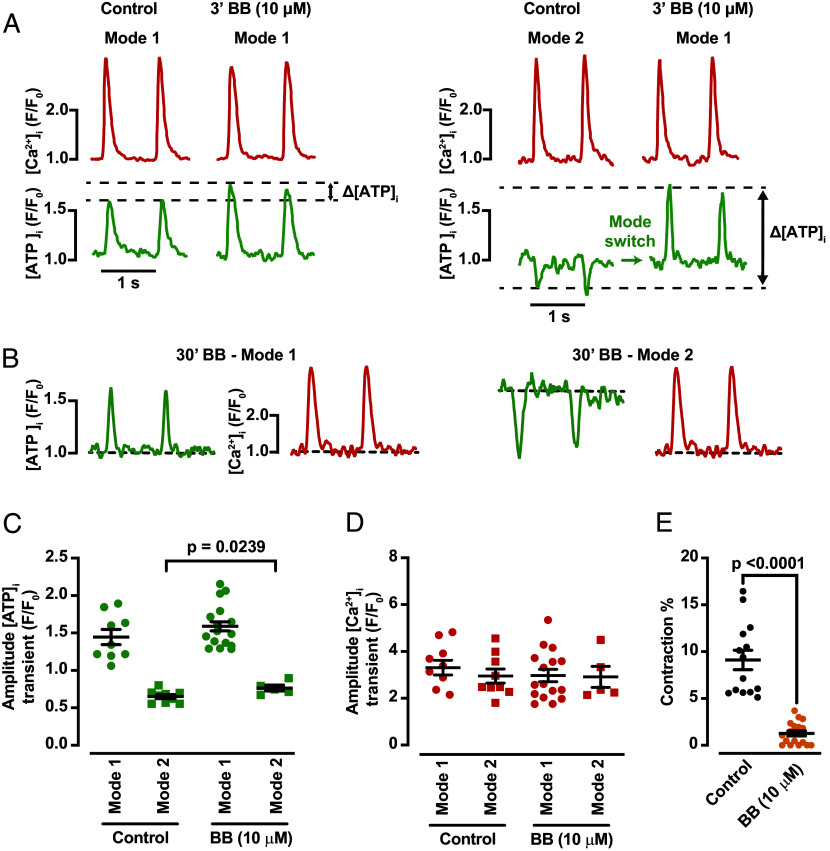
Contraction consumes significant ATP during cardiac EC coupling. (*A*) [Ca^2+^]_i_ and [ATP]_i_ transients recorded from the same cell sites before and after the acute (3 min) application of 10 μM blebbistatin (BB). (*B*) [Ca^2+^]_i_ and [ATP]_i_ transients recorded from cell sites in control cells and in cells exposed to 10 μM BB for 30 min. Scatter plots of the amplitude of [ATP]_i_ (*C*) and [Ca^2+^]_i_ (*D*) transients under control conditions and in the presence of 10 μM BB for 30 min (N = 4, n = 9/9 of control Mode 1 and Mode 2 sites, and N = 4, n = 16/5 of BB Mode 1 and Mode 2 sites, respectively). (*E*) Amplitude (%) of contraction in control cells (N = 4, n = 14) and cells treated with 10 μM blebbistatin (N = 4, n = 18). All significant values are provided from a nested *t* test. The mean values ± SEM of all individual values are in black.

A critical question raised at the outset of this study was what is the energetic cost of EC coupling in a ventricular myocyte? To answer this question, we imaged [Ca^2+^]_i_ and [ATP]_i_ before and after the application of blebbistatin (23), which has been shown to specifically inhibit actin–myosin interactions and hence contraction in these cells (Fig. 6).

Fig. 6*A* shows [Ca^2+^]_i_ and [ATP]_i_ records from representative myocytes displaying Mode 1 and Mode 2 Ca^2+^–ATP dynamics before and after the acute (∼3 min) application of blebbistatin (10 μM). Exposure to blebbistatin induced a subtle increase in the amplitude of the [ATP]_i_ transient in Mode 1 sites (Δ[ATP]_i_ = 0.23 ± 0.06 F/F_0_). Interestingly, in Mode 2 sites, blebbistatin treatment led to a positive [ATP]_i_ transient (Δ[ATP]_i_ = 0.51 ± 0.16 F/F_0_), effectively converting or “mode switching” this site into a Mode 1 site. However, blebbistatin did not change [Ca^2+^]_i_ in cells with Modes 1 and/or 2 sites.

Accordingly, chronic exposure to blebbistatin (i.e., ∼30 min), led to an increase of the percentage of cells with Mode 1 sites (i.e., 43% vs. 79%) and a decrease in Mode 2 sites (i.e., 57% vs. 21%) (Fig. 6 *B* and *C*). The amplitude of [ATP]_i_ transients in Mode 1 sites was moderately higher in chronic blebbistatin-treated myocytes than in controls (Fig. 6*C*). Conversely, the amplitude of Mode 2 iATP transients shifts from 0.67 under control conditions (i.e., a decrease of 0.33 F/F_0_ units) to 0.78 (i.e., a decrease of 0.22 F/F_0_ units) (*P* < 0.01). Because these are normalized values, the data mean that less ATP is being consumed at Mode 2 sites after blebbistatin treatment than in control conditions. Thus, we propose that the impact of the decrease in ATP consumption (an increase in Mode 1 sites and decrease in Mode 2 sites) in response to blebbistatin is to summate to generate a cell-wide increase in [ATP]_i_.

As with the acute application of blebbistatin, the drug did not alter [Ca^2+^]_i_ in cells with Modes 1 and/or 2 sites (Fig. 6*D*) but fully eliminated cell contraction (Fig. 6*E*). Taken together, these data suggest that cross-bridge cycling is a major consumer of ATP during EC coupling.

### Activation of β-Adrenergic Signaling Impacts Diastolic and Systolic [ATP]_i_.

Activation of β-adrenergic signaling pathways increases ATP consumption through several processes, such as cAMP production, SERCA pump activation, and protein phosphorylation (8, 24). Thus, we tested the hypothesis that application of the β-adrenergic receptor agonist isoproterenol (ISO) decreases diastolic [ATP]_i_ in ventricular myocytes. A representative time course of [ATP]_i_ changes before and after ISO (100 nM) treatment appears in *SI Appendix*, Fig. S9*A*. Consistent with our hypothesis, application of isoproterenol led to a decrease in diastolic [ATP]_i_ to a lower steady state (Δ[ATP]_i_ = 0.18 ± 0.05 F/F_0_; *P* < 0.01).

Next, we recorded [Ca^2+^]_i_ and [ATP]_i_ in control and isoproterenol-treated ventricular myocytes during pacing (1 Hz) and found that it increased global [Ca^2+^]_i_ (2.70 ± 0.18 F/F_0_ before and 3.46 ± 0.18 F/F_0_ after isoproterenol; *P* < 0.0001) and [ATP]_i_ (1.05 ± 0.02 F/F_0_ before and 1.13 ± 0.02 F/F_0_ after isoproterenol; *P* < 0.01) (*SI Appendix*, Fig. S9 *B* and *C*). Together, these data suggest that activation of β-adrenergic receptors increases diastolic ATP consumption, but this decrease in [ATP]_i_ is compensated during EC coupling likely because of increased Ca^2+^ entry and SR Ca^2+^ release during the AP.

### K_ATP_ Channels Are Active at the Diastolic Levels of [ATP]_i_ of Ventricular Myocytes.

**Fig. 7.**
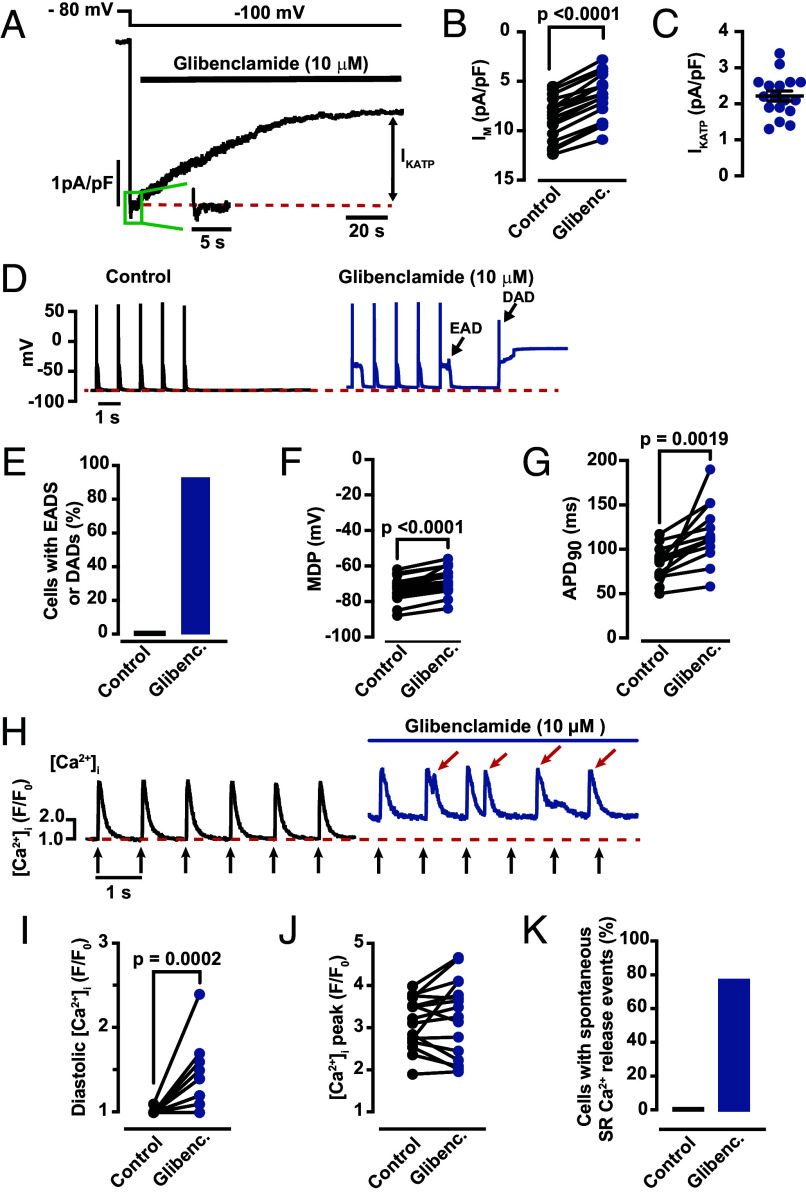
Active sarcolemmal K_ATP_ channels in ventricular myocytes. (*A*) Whole cell K^+^ currents recorded at −100 mV from a holding potential of −80 mV before and after 10 μm glibenclamide. The green *Inset* and dashed red line show the control current before drug. Scatter plots of current density before and after glibenclamide (*B*) and for the glibenclamide sensitive current (I_KATP_) (*C*) (N = 4, n = 17). (*D*) Current clamp recordings of AP-evoked in ventricular myocytes before and after application of glibenclamide. Black arrows note early and delayed after depolarizations (EADs or DADs, respectively) in the glibenclamide-treated cells. (*E*) Percentage of cells with recorded EADs or DADs. Scatter plots of membrane diastolic potential (MDP) (*F*) and the time to 90% of the action potential (APD_90_) (*G*) before and after glibenclamide (N = 4, n = 14). (*H*) AP-evoked (1 Hz) [Ca^2+^]_i_ transients in a ventricular myocyte before and after the application of 10 μM glibenclamide. The black arrows mark when an AP was evoked via field stimulation. The red arrows identified spontaneous Ca^2+^ release events. Scatter plots of diastolic [Ca^2+^]_i_ (*I*) and the amplitudes of [Ca^2+^]_i_ transients (*J*) under control conditions and in the presence of 10 μM glibenclamide (N = 4, n = 17). (*K*) Percentage of cells with spontaneous SR Ca^2+^ release events. All significant values are provided from a paired *t* test.

The final set of experiments were designed to investigate the physiological consequences of our data suggesting that diastolic [ATP]_i_ is nearly 500 μM (Fig. 7). One logical strategy to address this important issue would be to determine whether K_ATP_ channels are active at these [ATP]_i_ values. K_ATP_ channels are functionally expressed in ventricular myocytes and play a critical role in cardiac metabolic-excitation coupling. The open probability of the K_ATP_ channel is maximal at [ATP]_i_ < 1 μM and is nearly zero at concentrations > 2 mM (IC_50_ = 18 μM) (25), but can also be regulated via other mechanisms (26).

We began by recording membrane currents in ventricular myocytes before and after the application of the specific K_ATP_ channel antagonist glibenclamide (10 μM). For these experiments, we used the conventional configuration of the whole-cell patch-clamp technique using an internal solution with 500 μM ATP. During analysis, K_ATP_ currents were defined as the glibenclamide-sensitive component of the current at −100 mV. Fig. 7*A* shows a membrane current record from a representative ventricular myocyte held at −80 mV, which is near the Nernst equilibrium potential for K^+^ under our experimental conditions (−83 mV). Hyperpolarization to −100 mV elicited an inward current. The amplitude of this current was decreased by the application of glibenclamide (Fig. 7*B*). On average, the amplitude of the K_ATP_ current density was 2.2 ± 0.1 pA/pF (Fig. 7*C*).

Next, we recorded membrane voltages in ventricular myocytes before and after the application of 10 μM glibenclamide. The current clamp protocol involved the activation of APs with a small injection of current (1 nA) at a frequency of 1 Hz followed by a pause in stimulation. Fig. 7*D* shows membrane potential records from a representative ventricular myocyte. Consistent with the voltage-clamp data above, application of glibenclamide induced depolarization of the maximum diastolic potential as well as prolongation of the action potential duration at 90% repolarization (APD_90_). Notably, only 6% of cells had EADs or DADs under control conditions vs. 88% during K_ATP_ channel blockade (Fig. 7*E*). Indeed, a paired *t* test analysis showed that glibenclamide depolarized ventricular myocytes by 5.5 ± 2.2 mV (*P* < 0.0001) and APD_90_ increased by 34 ± 11 ms (*P* = 0.0019) (Fig. 7 *F* and *G*).

A third critical test of our hypothesis was to determine the impact of glibenclamide on intact myocytes, where ATP levels are not influenced in any way via a patch pipette. Accordingly, our iATP data suggest ATP levels are about 500 μM, therefore a fraction of K_ATP_ channels should be active and application of glibenclamide should increase the probability of spontaneous SR Ca^2+^ release in these intact myocytes.

Fig. 7*H* shows the results of a set of experiments in which we recorded AP-evoked [Ca^2+^]_i_ transients before and after the application of glibenclamide. Consistent with our hypothesis, we found that acute exposure of ventricular myocytes to 10 μM glibenclamide increased diastolic [Ca^2+^]_i_ (1.01 ± 0.01 F/F_0_ before and 1.35 ± 0.08 F/F_0_ after glibenclamide; *P* = 0.0002) (Fig. 7*I*) and the incidence of spontaneous SR Ca^2+^ release events. Indeed, 78% of the cells exposed to glibenclamide had spontaneous SR Ca^2+^ release events (Fig. 7*K*). In combination, our electrophysiological and [Ca^2+^]_i_ data are consistent with the observation that [ATP]_i_ is submillimolar and closer to where a subpopulation of K_ATP_ channels is active, likely hyperpolarizing and stabilizing diastolic membrane potential.

## Discussion

**Fig. 8.**
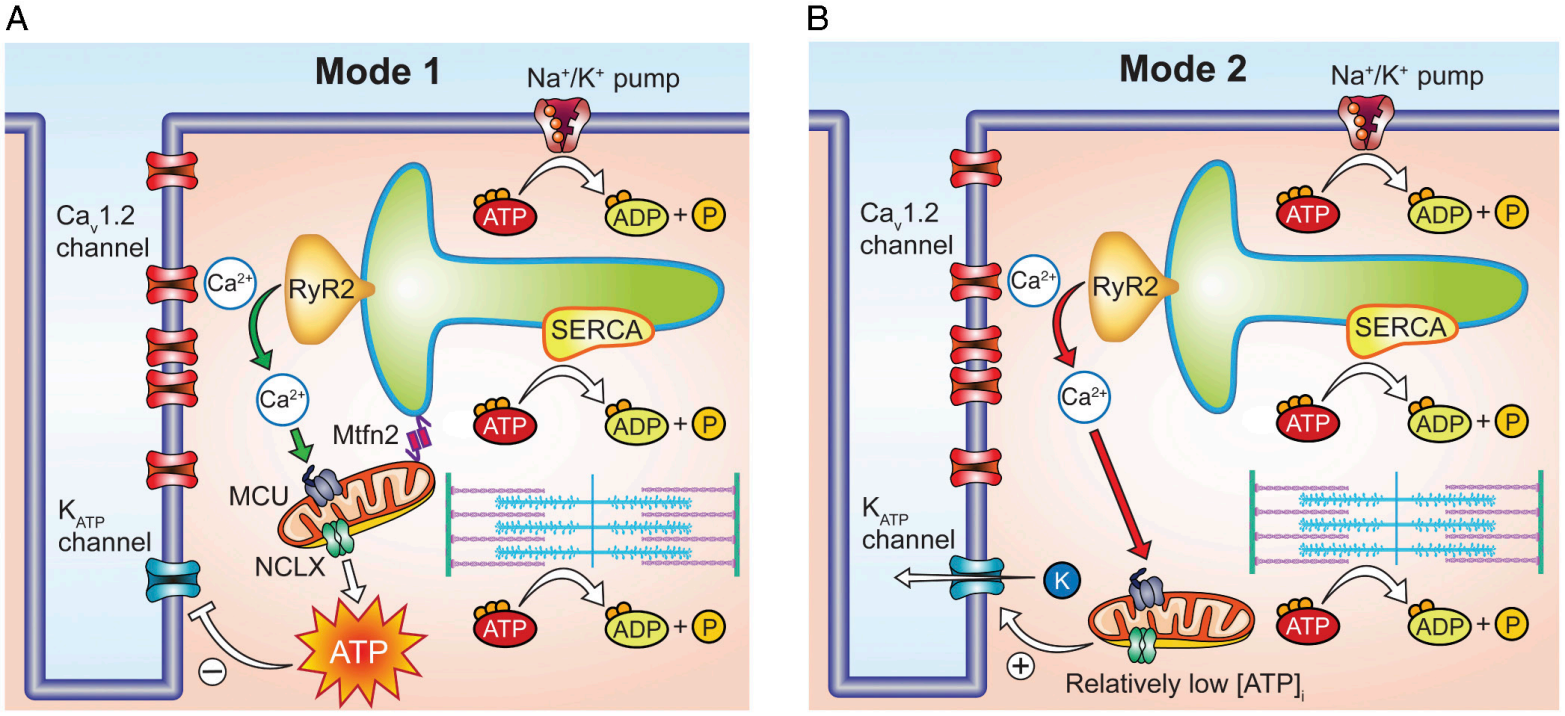
Proposed model for Modes 1 and 2 ATP dynamics in ventricular myocytes during EC coupling. (*A*) In Mode 1 sites, mitochondria, anchored to the SR by Mfn2, strategically positioned near sites of Ca^2+^ entry and release, efficiently absorb Ca^2+^ through the MCU, leading to ATP production. (*B*) In Mode 2 sites, mitochondria are likely located farther away from the dyad. Mitochondria in these sites initially experience a decrease in [Ca^2+^]_mito_, likely mediated by the NCLX. However, as Ca^2+^ diffuses to these mitochondria and enters via the MCU, this influx surpasses NCLX efflux, leading to an augmentation in [Ca^2+^]_mito_ that increases ATP synthesis and restores diastolic [ATP]_i_ levels in the vicinity.

In this study, we made three fundamental findings. First, we show that [ATP]_i_ is dynamically regulated during cardiac EC coupling. Second, we identified two distinct modes of [ATP]_i_ fluctuations, depending on the degree of SR-mitochondrial coupling. Third, contrary to the prevailing dogma that diastolic ATP levels in ventricular myocytes are very high (i.e., 8 to 10 mM), [ATP]_i_ are likely significantly lower (i.e., <1 mM), allowing a small population of K_ATP_ channels to be open under physiological conditions and hence control myocyte excitability at least during diastole. These findings lead us to propose a model for cardiac electrometabolic coupling that is diagrammatically represented in Fig. 8.

We identified two distinct modalities of [ATP]_i_ fluctuations during EC coupling. In Mode 1, activation of Ca^2+^ influx and SR Ca^2+^ release led to an elevation in [Ca^2+^]_mito_ and consequent ATP generation (Fig. 8*A*). Mitochondrial Ca^2+^ homeostasis is maintained by a balance between Ca^2+^ influx through the mitochondrial Ca^2+^ uniporter (MCU) (27–29) and efflux through the Na^+^/Ca^2+^ exchanger (NCLX). Mitochondria tethered to the SR by Mfn2 are advantageously located near sites of SR Ca^2+^ release. Intake of this Ca^2+^ (30) through the MCU causes an increase in [Ca^2+^]_mito_ that stimulates the TCA cycle to produce ATP (i.e., Mode 1 ATP dynamics). We propose that the physiological role of Mode 1 transients is to rapidly generate the ATP necessary to meet the energetic demands of EC coupling.

Conversely, Mode 2 was characterized by a net decrease in [ATP]_i_ during an AP (Fig. 8*B*). This decrease is likely caused by ATP hydrolysis by energy-consuming processes such as cross-bridge cycling and Ca^2+^ transport by the SR Ca^2+^ ATPase. Mitochondria in Mode 2 sites undergo an initial decrease in [Ca^2+^]_mito_, likely mediated by the NCLX. However, when Ca^2+^ from remote sites reach it and enters via the MCU, this Ca^2+^ influx surpasses NCLX efflux, augmenting [Ca^2+^]_mito_. This increases ATP synthesis and restores nearby diastolic [ATP]_i_.

Down-regulation of Mfn2 reduced the size of [ATP]_i_ transients in Mode 1 sites, emphasizing the role of Mfn2 in localizing mitochondria near dyadic regions (14–16). This positioning allows mitochondria to quickly produce ATP in response to Ca^2+^ entry and SR Ca^2+^ release, thereby activating the TCA cycle and increasing [ATP]_i_ (11–13).

Notably, Mfn2 expression has been shown to decrease during the development of heart failure (31). Based on this, it is intriguing to speculate that down-regulation of Mfn2 during this pathological condition would decrease SR-mitochondrial contacts and expression of coenzyme Q, which could lead to Mode 1-to-Mode 2 switching and thus lower ATP production. This is consistent with previous studies suggesting that Mfn2 has multiple functional roles in addition to promoting SR-mitochondrial tethering, including mitochondrial fusion/fission and maintaining coenzyme Q levels that impact substrate metabolism and ATP generation (14, 22, 32). Although coenzyme Q does not play a direct role in the TCA cycle, it serves as a crucial link between it and the electron transport chain in mitochondria. This may explain why down-regulation of Mfn2 decreased the slope [Ca^2+^]_mito_-[ATP]_i_ relationship in ventricular myocytes. Further investigation is warranted to test this hypothesis.

Our data have important physiological implications for electrometabolic coupling in ventricular myocytes. K_ATP_ channels play a key role in this process by coupling K^+^ flux, and hence excitability, to [ATP]_i_ in ventricular myocytes. The activity of endogenous cardiac K_ATP_ channels is maximal at [ATP]_i_ < 1 μM and nearly zero at concentrations >2 mM (IC_50_ ≈ 20 μM) (25). At ATP concentrations ranging from 300 to 800 μM, it is estimated that K_ATP_ channels in cardiac myocytes can reach ~2 to 6% of their maximum activity. Accordingly, most, if not all, existing models to date concerning ventricular myocyte excitability assume that K_ATP_ activity is zero. This presumption is based on the belief that cardiac ATP levels range from 8 to 10 mM and do not change under physiological conditions. However, in scenarios where ATP production is compromised, like during ischemic conditions, or when specific signaling pathways activating K_ATP_ channels are triggered, ventricular myocytes may undergo hyperpolarization (26). This effectively reduces excitability, contributing to energy conservation.

Our data suggest that diastolic [ATP]_i_ concentration is submillimolar and fluctuates during the AP, which challenges two central tenets of these long-held models. First, a subpopulation of K_ATP_ channels should be open during diastole. Consistent with this, we found that acute exposure of ventricular myocytes to the K_ATP_ channel antagonist glibenclamide increased the probability of spontaneous SR Ca^2+^ release events and DADs in ventricular myocytes, suggesting that K_ATP_ channels are open at diastolic [ATP]_i_ in mouse ventricular myocytes. Second, K_ATP_ channel activity likely changes during the AP, decreasing as ATP levels increase and/or increasing as ATP decreases.

Based on these data, we propose that [ATP]_i_ dynamics tune K_ATP_ channel activity during the different phases of the AP. In this view, K_ATP_ channel activity during diastole is not zero. Rather, a relatively small number of K_ATP_ channels are active, hyperpolarizing cells and decreasing the probability of spontaneous AP activation. However, during the AP the level of K_ATP_ activity will depend on the balance between Mode 1 and Mode 2 ATP sites within a ventricular myocyte. For example, in cells with multiple Mode 1 sites, the overall K_ATP_ channel activity likely decreases as [ATP]_i_ increases, minimizing the impact of these channels on AP duration. The opposite would apply to cells in which Mode 2 [ATP]_i_ dynamics dominate. Because ~85% of myocytes exhibited increases in [ATP]_i_ during EC coupling, K_ATP_ activity should be minimal during the AP of most cells. Future experiments should test these provocative hypotheses.

An interesting finding in this study is that while the magnitude of contraction is similar in cells regardless of the type of their ATP fluctuation modalities, the time-to-peak of contraction in cells with elevations in ATP (i.e., Mode 1) was faster than cells with net decreases in ATP (i.e., Mode 2 sites). These data are consistent with classic studies suggesting that while isometric tension is not a strong function of ATP concentration in the range 50 μM to 1 mM, the maximum velocity of contraction increases up to 1 mM ATP (33, 34). Thus, fluctuations in [ATP]_i_ observed here during EC coupling may introduce a unique regulatory mechanism for contraction.

In line with earlier work by Gibbs et al. (35, 36), our findings indicate that ATP consumption during EC coupling primarily stems from cross-bridge cycling. Moreover, our data align with studies in neurons (37), suggesting that the action potential’s role in ATP consumption during cardiac EC coupling is relatively minor. However, inhibition of mitochondrial ATP synthase or oxidative phosphorylation led to a gradual decrease in intracellular ATP in dormant ventricular myocytes, highlighting the substantial contribution of basal processes like ion transport and protein expression to ATP consumption over longer time scales.

We found that isoproterenol decreased diastolic [ATP]_i_. This is likely due to the activation of multiple signaling events. For example, increases in cAMP production by adenyl cyclases (AC) and the subsequent activation of protein kinase A (PKA) during β-adrenergic signaling consume ATP (8). PKA-driven increases in the activity of SERCA pumps and cross-bridge cycling is also expected to increase ATP consumption (24). However, the observation that activation of β-adrenergic signaling increases the amplitude of the [Ca^2+^]_i_ and the [ATP]_i_ transients during EC coupling is consistent with the hypothesis that increased Ca^2+^ entry and SR Ca^2+^ likely enhances ATP production, compensating for the increase in ATP consumption, which ultimately leads to a net [ATP]_i_ increase during the AP.

The activity of ACs depends on [ATP]_i_, but their ATP requirements differ among isoforms. For example, the Michaelis constant (K_m_) of AC5 and AC6 for ATP is about 20 μM, which suggests that with [ATP]_i_ ranging from about 300 to 800 μM these enzymes would be operating at high or near maximal rates. The K_m_ of AC9 for ATP under basal conditions is similar (~5 to 11 μM) but increases to >800 μM during G_αs_ activation (38), which is relatively close to the estimated levels of diastolic [ATP]_i_. This is important because it suggests that the rate of cAMP production may change during the AP as ATP levels change.

AC9 is not the only signaling molecule that could be subjected to regulation by ATP fluctuations between 300 and 800 μM. For example, the K_m_ of PI_4_ kinase for ATP is ∼0.4 to 1 mM (9, 39–41). This suggests that ATP fluctuations may represent a mechanism of cAMP and PIP_2_ regulation within a ventricular myocyte in a beat-to-beat fashion. Similarly, it would be interesting to investigate whether deficits in ATP production during pathology are associated with changes in these signaling pathways, as suggested for PIP_2_ in capillary endothelial cells during small vessel diseases (42). Indeed, it would be interesting to determine [ATP]_i_ using expressible indicators in all cell types in the cardiovascular system, including endothelial and smooth muscle cells.

RyR2s are also subject to regulation by ATP - the EC_50_ of RyR2s for ATP is approximately 200 μM (43). Thus, a plethora of intracellular processes including cAMP and PIP_2_ levels as well as SR Ca^2+^ release could be subjected to regulation by the changes in ATP levels reported here.

While our findings seemingly deviate from previous studies suggesting that ATP levels in whole hearts range from 8 to 10 mM, our work does not directly address the mechanistic basis of this discrepancy. An intriguing hypothesis is that previous studies measured total ATP levels, including bound and stored forms, whereas our iATP measurements reflect only free cytosolic ATP concentrations. Thus, analogous to Ca^2+^, a significant portion of ATP may be bound to proteins or sequestered within intracellular stores, potentially accounting for the higher total ATP levels observed in whole heart preparations. Further investigations are warranted to elucidate the mechanistic basis underlying these divergent ATP measurements and to comprehensively characterize the distribution and dynamics of ATP across subcellular compartments.

To conclude, we propose a model in which [ATP]_i_ is subject to dynamic regulation in ventricular myocytes during EC coupling. We identified two distinct modes of ATP fluctuations, depending on the degree of SR-mitochondrial coupling. These findings compel modification of long-standing models of excitation-metabolic coupling as well as other signaling pathways that are modulated by ATP in cardiac muscle.

## Materials and Methods

A detailed version of this study’s materials and methods can be found in *SI Appendix*. Briefly, wild-type C57BL/6 J mice (8 to 12 wk old) were used in this study. All datasets are normally distributed. Hierarchical (nested) statistical analyses or paired Student’s *t* test were implemented throughout the paper.

## Supplementary Material

Appendix 01 (PDF)

## Data Availability

All study data are included in the article and/or *SI Appendix*.
