# Supplementary material for: Fueling the heartbeat: Dynamic regulation of intracellular ATP during excitation–contraction coupling in ventricular myocytes
Source: Proc Natl Acad Sci U S A. 2024 Jun 12;121(25):e2318535121. doi: 10.1073/pnas.2318535121 (PMC11194497; doi:10.1073/pnas.2318535121)
Supplement: Supplementary file 1 — Appendix 01 (PDF) [file pnas.2318535121.sapp.pdf]

PNAS
www.pnas.org

Supplementary Information (SI) for

Fueling the Heartbeat: Dynamic Regulation of Intracellular ATP During Excitation-Contraction Coupling in Ventricular Myocytes

Paula Rhana, Collin Matsumoto, Zhihui Fong, Alexandre D. Costa, Silvia G. Del Villar, Rose E. Dixon, and L. Fernando Santana

Department of Physiology & Membrane Biology, School of Medicine, University of California, Davis

***Correspondence to:**
L. Fernando Santana (lfsantana@ucdavis.edu)

This PDF file includes:

Supplementary text
Figures S1 to S9
SI References

Supplementary Information Text

Materials and Methods

Infection of mice with adeno-associated viruses

This study utilized male C57BL/6J mice aged 8-12 weeks. Adeno-associated virus serotype 9 (AAV9) vectors containing the genetically encoded iATP sensor (iATPSnFR$^{1.0}$) (1) were prepared at a concentration of 4 x 10^{12} viral genome copies per milliliter (vg/mL). To perform the retro-orbital infection, mice were anesthetized with 5% isoflurane, and 100 µL of the AAV9 vector solution was injected into the retro-orbital sinus of each mouse using a micro syringe. For the knockdown of mitofusin 2, AAV9 vectors containing the mitofusin 2-shRNA (UC Davis Cardiovascular Research Institute Viral Vector core) or a scramble sequence (Vector Biolabs, USA) were prepared at a concentration of 4 x 10^{12} vg/mL and retro-orbitally injected. All experimental procedures were conducted following the guidelines of the University of California, Davis Institutional Animal Care and Use Committee.

Isolation of mouse ventricular myocytes

Animals were euthanized 1-2 weeks post infection by administering a lethal dose of sodium pentobarbital via intraperitoneal injection. The heart was excised, the aorta was cannulated and then mounted in a Langendorff system for cardiomyocyte isolation, following the enzymatic assay previously described by Shioya (2). Isolated ventricular myocytes were maintained at room temperature (24°C) in Tyrode's solution (in mM: 130 NaCl, 5.4 KCl, 1.8 $CaCl_2$, 0.33 NaH_2PO_4, 25 HEPES, 22 glucose, and 0.5 $MgCl_2$, pH adjusted to 7.4 using NaOH) until used. Experiments were conducted up to 5 hours after isolation.

Patch-clamp electrophysiology

All electrophysiological recordings were performed using an Axopatch 200A amplifier and Clampex 10 software (Molecular Devices, USA). Membrane currents and voltages were acquired at a sampling frequency 10 kHz. All recordings were performed at physiological 35-37 °C. Membrane currents, maximum diastolic potentials, and action potential durations were measured using Clampfit (Molecular Devices, USA).

Ionic currents were recorded from ventricular myocytes using the whole-cell configuration of the patch-clamp technique. The internal solution used for voltage-clamp recordings contained (in mM): 140 KCl, 5 NaCl, 0.5 $MgCl_2$, 11 EGTA, 0.5 ATP-Mg and 10 HEPES (pH = 7.2 with KOH). Micropipettes filled with this solution had a resistance ranging from 0.8 to 1.5 MΩ. Myocytes were first patched in a Tyrode's solution. After successful conversion to the whole-cell configuration of the patch-clamp technique, voltage-clamped myocytes were exposed to an external solution containing (in mM): 140 NMDG, 5.4 KCl, 1.8 $CaCl_2$, 0.5 $MgCl_2$, 0.33 NaH_2PO_4, 5 Glucose, 11 HEPES and 0.1 $CdCl_2$ (pH to 7.4 with KOH). Voltage-clamped cells were held at -80 mV, which is near the Nernst equilibrium potential (E_K) for K^+ (i.e., -83 mV) under our experimental conditions. During recordings, cells were hyperpolarized to -100 mV while being perfused with the NMDG-based external solution. After a stable steady-state currents were recorded, myocytes were exposed to a solution containing the ATP-sensitive K^+ (K_{ATP}) channel antagonist glibenclamide (10 µM). K_{ATP} currents were defined as the glibenclamide-sensitive component of the total currents.

The internal solution used for current-clamp recordings contained (in mM): 130 L- Aspartic acid, 5 NaCl, 1 $MgCl_2$, 5 EGTA, 0.5 ATP-Mg, 10 phosphocreatine and 10 HEPES (pH = 7.2 with KOH). Myocytes were patched in Tyrode's solution. After breaking into cell and and attaining the whole-cell configuration of the patch-clamp technique, the amplifier was set to current clamp mode. Myocytes were subjected to a protocol in which trains of action potentials (10X) were evoked by a small (1 nA) and brief (1 ms) injection of current at a frequency of 1 Hz.

Field stimulation

To induce action potentials in isolated ventricular myocytes, field stimulation was applied using two platinum wires (0.5 cm separation) positioned at the bottom of the perfusion chamber. A Grass stimulator (AstroMed Inc., USA) was employed to generate square voltage pulses with a duration of 4 ms. These pulses had an amplitude of 10-40 V and were delivered at a frequency of 1.0 Hz.

Loading of mitochondria with dh-Rhod-2

To monitor mitochondrial Ca^{2+} ($[Ca^{2+}]_{mito}$), ventricular myocytes were loaded with the mitochondrial Ca^{2+} indicator dh-Rhod-2 (Thermo Fisher, USA) as described previously (3). Briefly, a 1 mM stock solution of Rhod-2 was reduced using Na^+ borohydride, resulting in the formation of dh-Rhod-2. Isolated ventricular myocytes were then loaded with 5 μM dh-Rhod-2 solution for 30 minutes at 37 ^oC promoting compartmentalization of the indicator within the mitochondria. To facilitate de-esterification, myocytes were washed in Tyrode's solution before the imaging procedure.

Laser scanning confocal microscopy

We used an Olympus FV3000 (Olympus, Japan) laser scanning microscope for many of the experiments described in this paper. Fluorescence molecules were excited with solid state lasers emitting 488 nm or 561 nm light through a 60× oil-immersion lens (PlanApo) with a numerical aperture = 1.40. All confocal images were subject to background subtraction and analyses using ImageJ.

In-situ iATP calibration

Two complementary approaches were used to determine the relationship between ATP and iATP fluorescence in ventricular myocytes. The first approach involved the use of β-escin-permeabilized myocytes. For these experiments, acutely isolated ventricular myocytes expressing the iATP sensor were incubated with 2-deoxyglucose for 30 minutes at 37 ^oC and a droplet of myocytes was then added to a temperature-controlled chamber on the stage of the microscope containing Ca^{2+}- and glucose-free Tyrode's solution. After cells were allowed to settle at the bottom of the chamber for 5 minutes, they were permeabilized with β-escin (0.01 mg/ml). Confocal images were collected while cells were sequentially perfused with a solution containing and 0.1, 0.5, 2.5, 5 or 10 mM ATP.

For the second strategy, we used the whole cell configuration of the patch clamp technique to dialyze ventricular myocytes expressing the iATP sensor with varied ATP concentrations. Confocal images were collected before and after breaking into a cell with a micropipette (1–2 MΩ) filled with an internal solution containing (in mM): 130 aspartic

acid, 5 NaCl, 1 MgCl2, 5 EGTA, 10 HEPES, 10 phosphocreatine, and 0.1, 0.5, 1.0, 2.5, 5 or 10 ATP (pH = 7.2).

Images were analyzed using ImageJ. After background subtraction, ROIs were selected within each cell and fluorescence signals were normalized by dividing the difference between the fluorescence at each point (F) and the basal fluorescence (F_0) by F_0. The $[ATP]_i$-iATP fluorescence relationship was fit with a Hill equation:

$$((F - F_0)/F_0) = B_{max} \times [ATP]^h/((K_D^h + [ATP]^h))$$

Where B_{max} is the maximum value, h is the Hill coefficient, and K_d the apparent dissociation constant.

$[Ca^{2+}]_i$, $[Ca^{2+}]_{mito}$, and $[ATP]_i$ confocal imaging

Ventricular myocytes expressing the iATP sensor were loaded with the red-fluorescent acetoxymethyl-ester form of the cytosolic Ca^{2+} ($[Ca^{2+}]_i$) indicators Rhod-3 (10 μM). dh-Rhod-2 loading was performed as described above.

During experiments, a drop of myocytes was then added to a temperature-controlled chamber on the stage of the microscope containing Tyrode's solution. Cells were allowed to settle at the bottom of the chamber for 5 minutes before field stimulation was initiated. All experiments were performed at 37°C. Action potential-evoked $[Ca^{2+}]_i$, $[Ca^{2+}]_{mito}$, and $[ATP]_i$ signals were simultaneously acquired from the same confocal volume with the Olympus FV3000 operating in the line-scan mode (2 ms/line). iATP sensor and dh-Rhod-2 or Rhod-3 were excited with a 488 nm and 561nm laser light, respectively. Autofluorescence corrected and background subtracted fluorescence signals were normalized by dividing fluorescence at each point (F) with the baseline fluorescence (F_0) using ImageJ.

iATP fluorescence signals were converted to concentration units using the "F_{max}" equation (4):

$$[ATP] = K_d \ (F / (F_{max} - 1 / R_f))/(1 - F / F_{max})$$

Where F is fluorescence, F_{max} is the fluorescence intensity of iATP sensor in the presence of a saturating ATP concentration (in our case 10 mM), K_d is the dissociation constant of the fluorescence indicator used ($iATPSnFR^{1.0}$ = 1460 μM), and R_f is the indicator's dynamic range (3.8). The K_d and R_f values we used were empirically determined in ventricular myocytes as described above.

Rhod-3 fluorescence values were converted to concentration units using the pseudo ratio method (5). A K_d of 720 nM for Rhod-3 (6) and a resting level of $[Ca^{2+}]_i$ of 150 nM (7) was used in the calculations.

Super-resolution radial fluctuations (SRRF)

Acutely isolated ventricular myocytes expressing the iATP sensor were loaded with dh-Rhod2 and 250 nM Mitotracker Deep Red (Molecular Probes, USA) for 30 minutes at 37°C. Super-resolution radial fluctuations (SRRF) imaging was collected via Fusion software on an Andor Dragonfly 200 spinning disk confocal system (Andor Technologies, UK) coupled to a DMi* Leica microscope (Leica, Germany) equipped with a 60x oil

immersion objective (NA = 1.40) and acquired using an Andor iXon EMCCD camera. Single plane images were analyzed using ImageJ. iATP, dhRhod2 and Mitotracker Far Red signals were background subtracted. Colocalization based on the Pearson coefficient of iATP to Mitotracker and dhRhod2 to Mitotracker were measured using JACoP plugin in ImageJ (Bolte and Cordelieres, Journal of Microscopy, 2006).

Fluorescence recovery after photobleaching

Local fluorescence recovery after photobleaching experiments were performed using our Olympus FV3000 confocal laser-scanning microscope. Two-dimensional baseline images were acquired every 2 seconds. We used high-intensity 405 nm laser light (100% laser for 1 second) to photobleach iATP in subcellular regions of about 4 μm in diameter using the "tornado" bleaching mode of the microscope. After bleaching, iATP fluorescence was monitored for least 5 minutes under baseline imaging conditions to track recovery.

To determine the diffusion co-efficient of iATP, we first normalized the iATP recovery fluorescence to the average iATP signal from the baseline, pre-bleaching images. We then fit the normalized fluorescence recovery signal with the following function:

$$Y = Y\_0 + SpanFast(\ 〚1 - e〛\ \text{^}(-Kfast * X))\ +\ SpanSlow(\ 〚1 - e〛\ \text{^}(-Kslow * X))$$
$$SpanFast\ =\ (Plateau - Y\_0) \times PercentFast\ x\ 0.01$$
$$SpanSlow\ =\ (Plateau - Y\_0) \times (100 - PercentFast)\ x\ 0.01$$

where *X* is time, *Y* is normalized fluorescence, Y_0 and *plateau* are the same units as *Y*, K_{fast} and K_{slow} are rate constants in units reciprocal of time and *Percent fast* is the percent of signal due to the fast phase. We then used the approach of Axelrod *et al.* (8) to determine the diffusion coefficient.

$$D = 0.88 \times r\text{^}2/\ 〚4T〛\_(1/2)$$

where D is the diffusion coefficient, r is the radius in μm of the bleached ROI and $T_{1/2}$ determined from the exponential fit.

Reverse Transcriptase (RT) and Quantitative PCR

Total RNA was isolated using the RNeasy Mini Kit (Qiagen, Germany) as per manufacturer's instructions. Isolated mRNA from dissected ventricular tissue was then reverse transcribed using the AffinityScript qPCR cDNA Synthesis Kit (Agilent, USA) following manufacturer's protocol.

Synthesized cDNA was used to perform both RT-PCR and quantitative PCR (qPCR). Specific primers were designed complimentary to each target sequence of interest, including **β-actin** (NM_007393.5): sense nt (895-914): CCAGCCTTCCTTCTTGGGTA, antisense nt (989-967): AGAGGTCTTTACGGATGTCAACG; **mitofusin 1** (NM_024200.5): sense nt (1106-1126): AAAGCATAAAGCTCAGGGGAT, antisense nt (1356-1336): GGTCTTCCCTCTCTTCCATTG; and **mitofusin 2** (NM_001285920.1): sense nt (1285-1303): AGTACATGGAGGAGGTGCG, antisense nt (1522-1503): TCCTCAAACTGCCTCTCGAA.

Chemicals

All chemical reagents were acquired from Sigma-Aldrich (USA) unless otherwise stated.

Statistics

Data are expressed as mean ± standard error of the mean (SEM). All data sets were subjected to a Shapiro-Wilks normality test and passed it ($p > 0.05$). Accordingly, only parametric statistics were implemented. Hierarchical statistics (e.g., nested *t*-tests) and paired Student's *t*-test were used throughout the paper, as indicated in the figure legends. ANOVA analyses were followed by multiple comparison tests (i.e., Tukey). $P < 0.05$ was considered statistically significant.

Figures and Tables

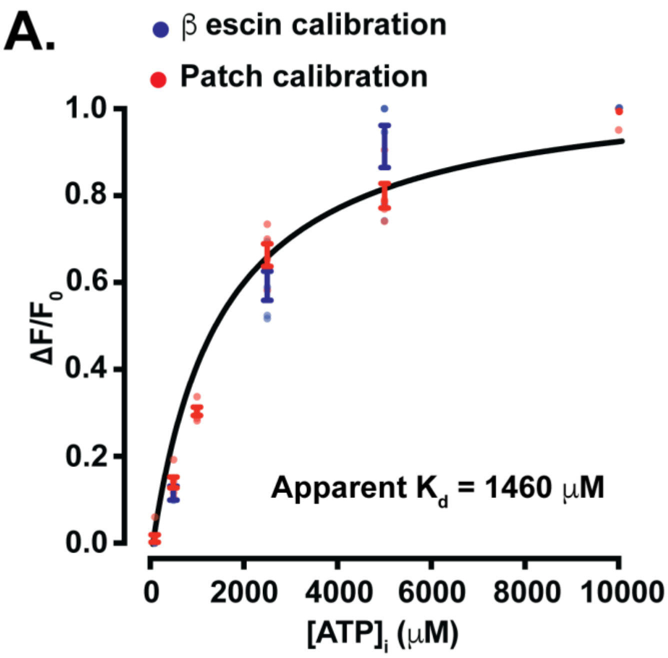

SI appendix Figure S1. Sub-millimolar diastolic $[ATP]_i$ in mouse ventricular myocytes. (A) Relationship between normalized iATP fluorescence and $[ATP]_i$ from β-escin treated (blue, N = 4, n = 6) and patched cells (red, N = 3, n = 7 / 6 / 5 / 5 / 5 / 6) for 100, 500, 1000, 2500, 5000 or 10000 μM ATP-Mg. Black solid line is a best fit (R^2 = 0.95) of both data sets with a single Hill equation as described in Materials and Methods section (i.e.,$\frac{\Delta F}{F_0} = B_{max} \times \frac{[ATP]^h}{(K_d^h + [ATP]^h)}$) using a least-squares routine (B_{max} = 1, apparent K_d = 1460 μM, , and h = 1). **(B)** Scatter plot of diastolic $[ATP]_i$ in μM units (N = 7, n = 18).

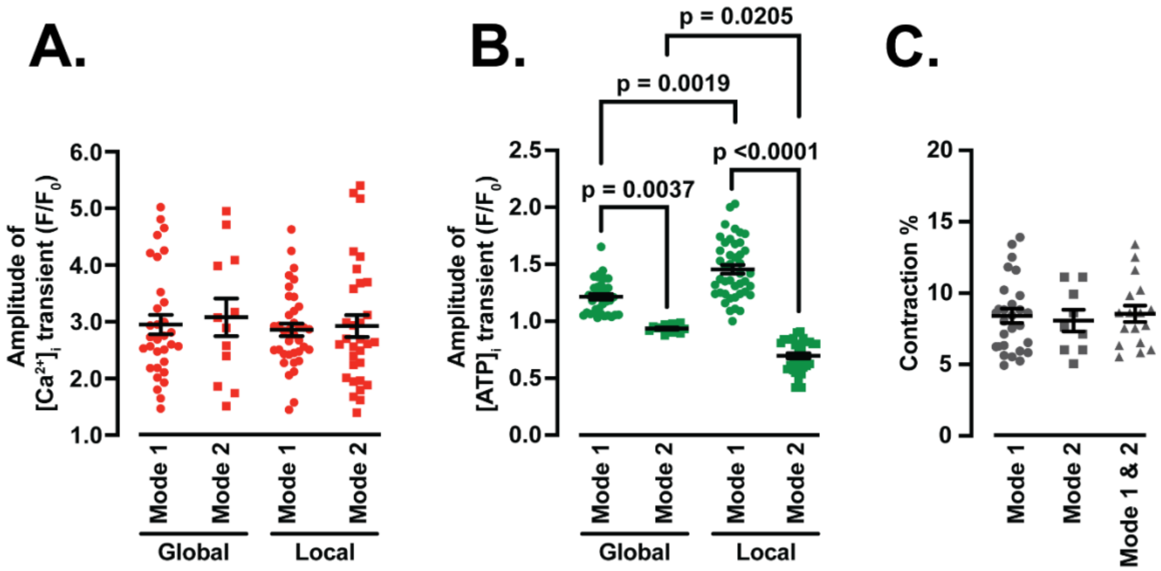

SI appendix Figure S2. **The amplitude of $[ATP]_i$ transients, but not $[Ca^{2+}]_i$ transients or contraction, varies between myocytes**. Scatter plots of the amplitudes of $[Ca^{2+}]_i$ (**A**) and $[ATP]_i$ (**B**) global and local transients (N = 12, n = 32 and N = 9, n = 12 of global Mode 1 and Mode 2, respectively; N = 13, n = 37 and N = 13, n = 31 for local Mode 1 and Mode 2, respectively). (**C**) Contraction amplitude (%) of ventricular myocytes with Mode 1 (N = 11, n = 26), Mode 2 (N = 7, n = 9), and Mode 1 & 2 (N = 8, n = 17) $[ATP]_i$ transients. All significant values are provided from a nested *t*-test. The mean values ± SEM of all individual values are in black.

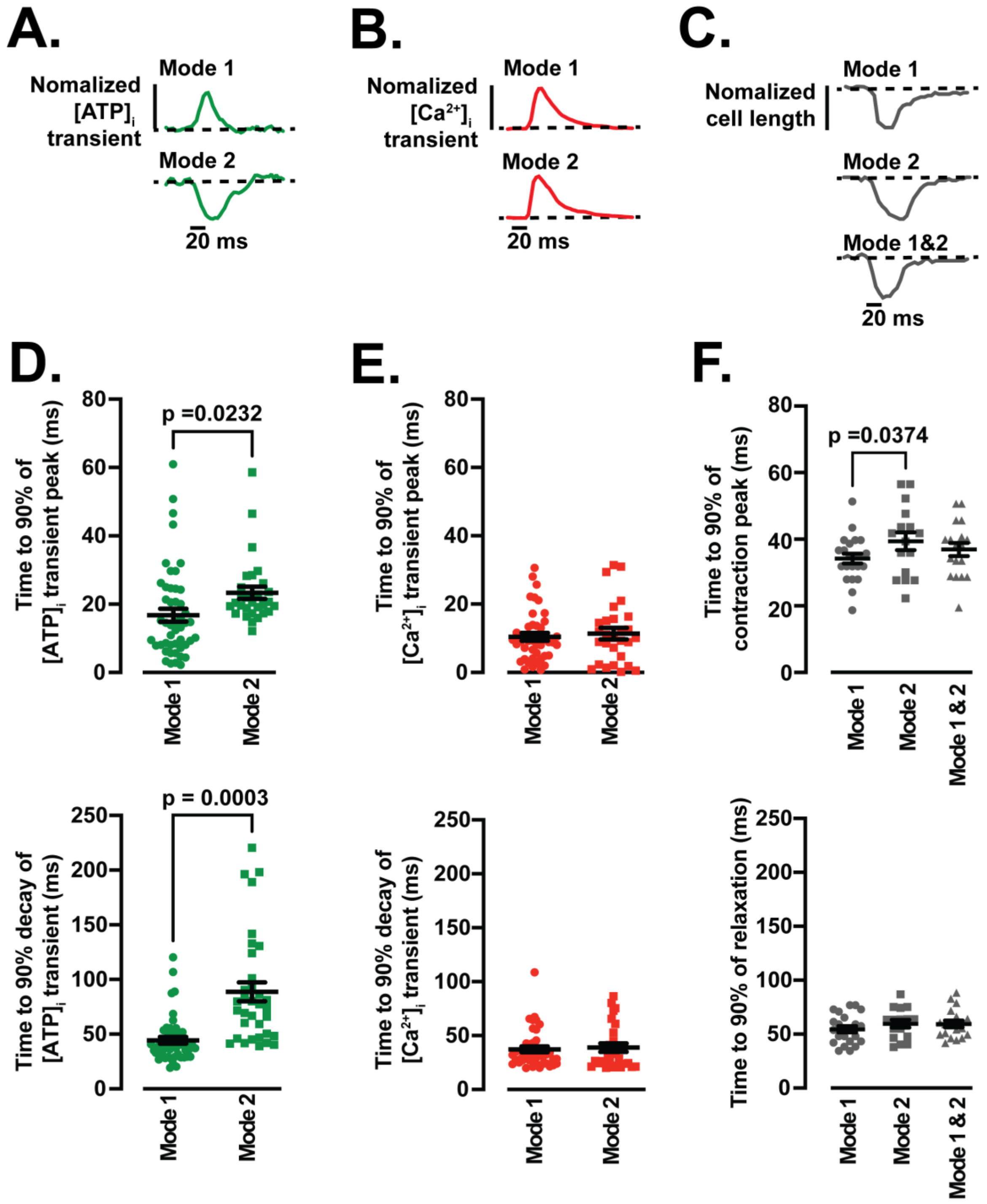

SI appendix Figure S3. Analysis of the kinetics of $[ATP]_i$, $[Ca^{2+}]_i$, and contraction. Time course of $[Ca^{2+}]_i$ (**A**), $[ATP]_i$ (**B**), and cell length (**C**) from Mode 1, 2, or 1 & 2 sites in representative cells**.** Scatter plots of the time to 90% of the amplitude (top) and decay (bottom) of $[Ca^{2+}]_i$ transients (**D**, N = 15, n = 42 / 28 of Mode 1 and Mode 2 sites), $[ATP]_i$ transients (**E**, N = 15, n = 47 / 29 of Mode 1 and Mode 2 sites), and contraction (**F**, N = 15**,** n = 22 / 16 / 18 of Mode 1, Mode 2 and Mode 1 & 2 cells). All significant values are provided from a nested *t*-test. The mean values ± SEM of all individual values are in black.

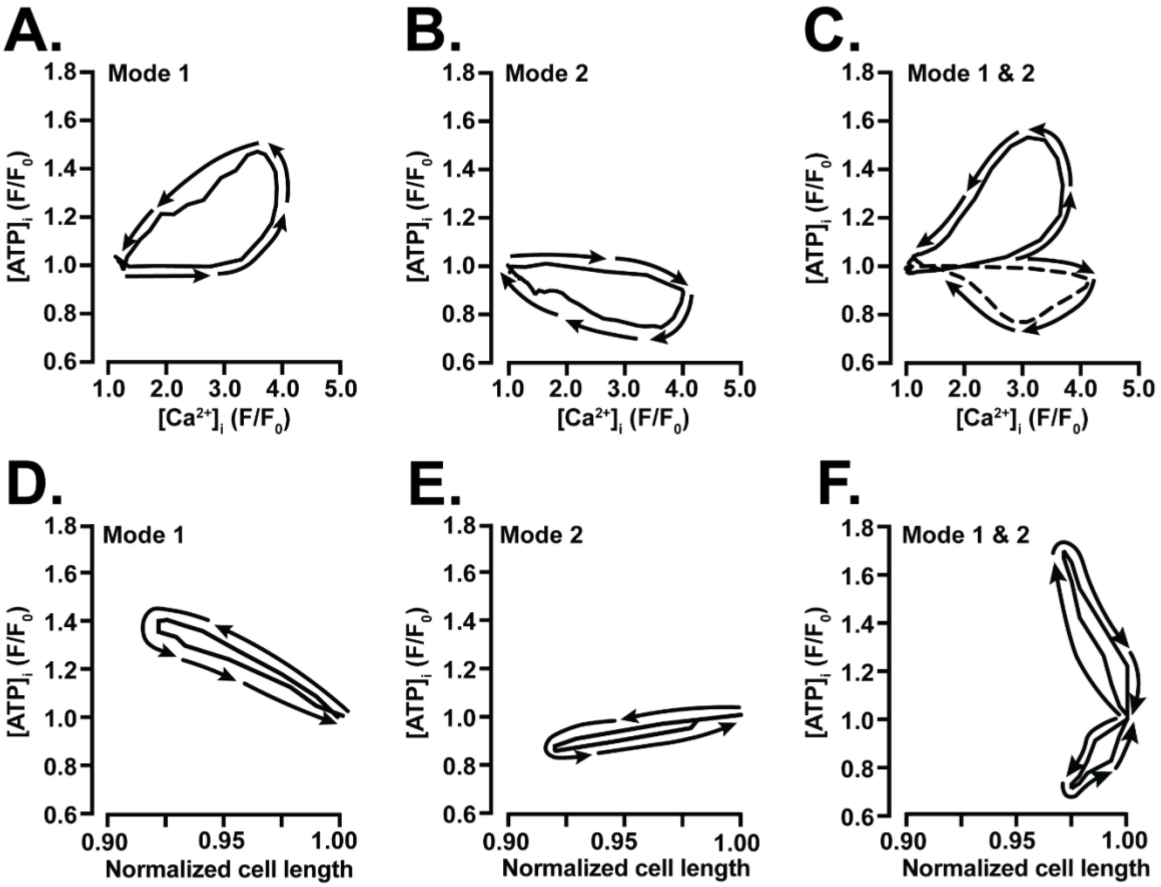

SI appendix Figure S4. Relationship between $[Ca^{2+}]_i$, $[ATP]_i$, and cell shortening during cellular contraction. Panels **A, B,** and **C** show the relationship between $[ATP]_i$ and $[Ca^{2+}]_i$ during the action potential plotted as a trajectory in cells displaying Mode 1, Mode 2, or Mode 1&2 ATP dynamics, respectively. Panels **D**, **E**, and **F** show the relationship between contraction and $[ATP]_i$. For each trace, the cells begin and end in a fully relaxed state at low $[ATP]_i$ and $[Ca^{2+}]_i$. Thus, the diagrams "begin" and "end" at the intersection of 1.0 in the x- and y- axes.

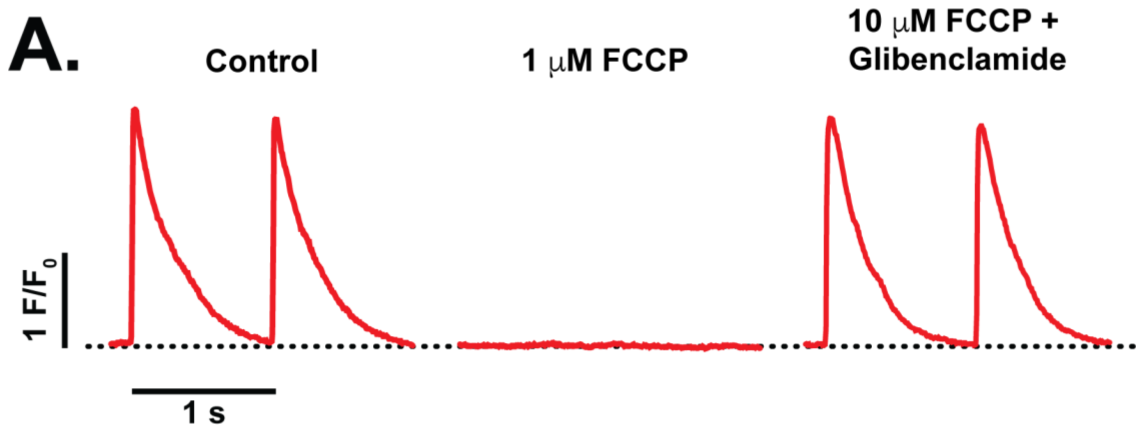

SI appendix Figure S5. Blockage of K_{ATP} channels restores EC coupling in ventricular myocytes. (A) Action potential-evoked global $[Ca^{2+}]_i$ transients from a representative ventricular myocyte under control conditions and the after application of the mitochondrial oxidative phosphorylation uncoupler FCCP (1 μM) alone or with the K_{ATP} blocker glibenclamide (10 μM).

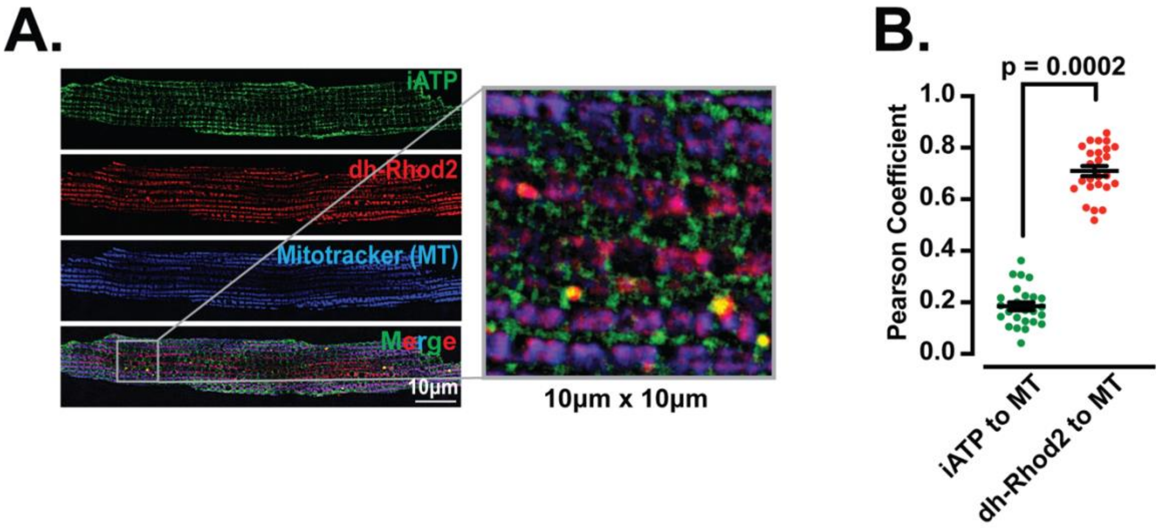

SI appendix Figure S6. iATP is cytosolic. (A) Representative single plane super resolution radial fluctuations (SRRF) imaging of a live ventricular myocyte expressing iATP (green) and loaded with dh-Rhod2 (red) and Mitotracker Far Red (Blue). Inset provides 10 x 10 μm merged image. **(B)** Scatter plots of the Pearson colocalization coefficient for iATP/Mitotracker (green) and dh-Rhod2/Mitotracker (red) (N = 3, n = 25). All significant values are provided from a nested *t*-test. The mean values ± SEM of all individual values are in black.

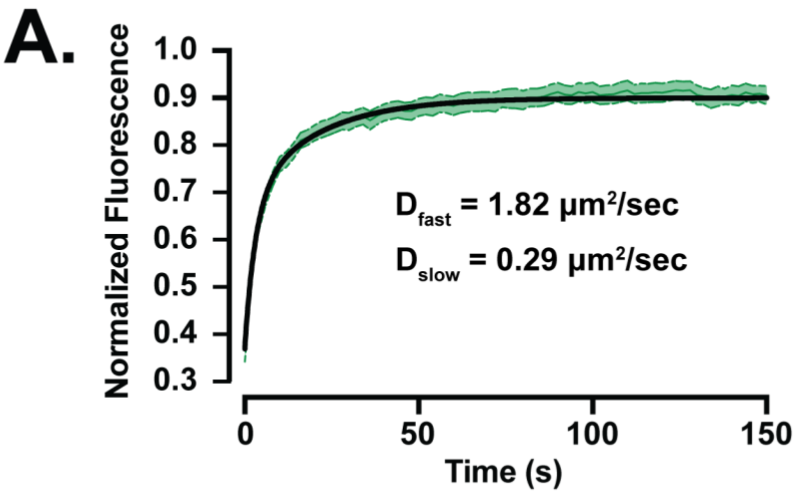

SI appendix Figure S7. iATP is mobile within the cytoplasm. (A) Time course of fluorescence recovery within a photobleached (FRAP) region of interest (ROI). Averaged traces are presented in green with opaque green shaded region indicating SEM (N = 3, n = 18). iATP recovery was fit with a two-phase association equation (see Materials and Methods section) overlayed in black with an average τ_{fast} of 3.12, $t_{1/2,fast}$ of 2.16, τ_{slow} of 19.65, $t_{1/2,slow}$ of 13.62 and ROI radius of 4.23 µm. The calculated diffusion coefficient for fast and slow components are 1.82 µm^2/sec and 0.29 µm^2/sec, respectively.

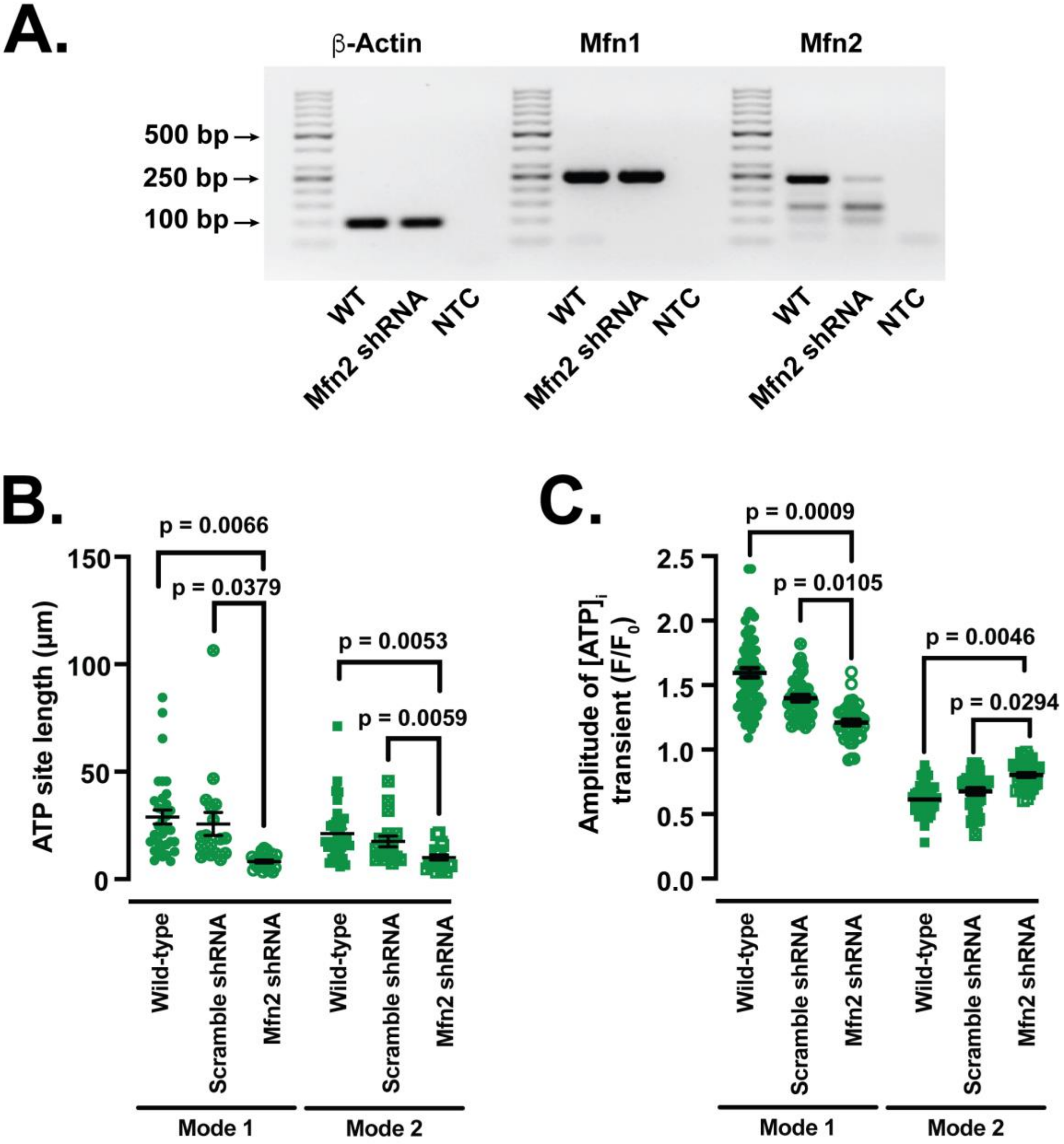

SI appendix Figure S8. Down-regulation of Mfn2 in cardiac muscle. (A) RT-PCR showing expression of β-actin, Mfn1, and Mfn2 transcripts in hearts from wild-type (WT) mice and hearts from mice injected with an adeno-associated virus serotype 9 (AAV9) expressing Mfn2-shRNA. Scatter plots of ATP site length (**B**, N = 7, n = 33; N = 3, n = 18 and N = 3, n = 23 for WT, scramble and Mfn2 shRNA, respectively) and $[ATP]_i$ transient amplitude (**C**, N = 10, n = 44; N = 4, n = 33 and N = 6, n = 42 for WT, scramble and Mfn2 shRNA, respectively) in wild-type, scramble shRNA and Mfn2 shRNA myocytes. All significant values are provided from a nested *t*-test. The mean values ± SEM of all individual values are in black.

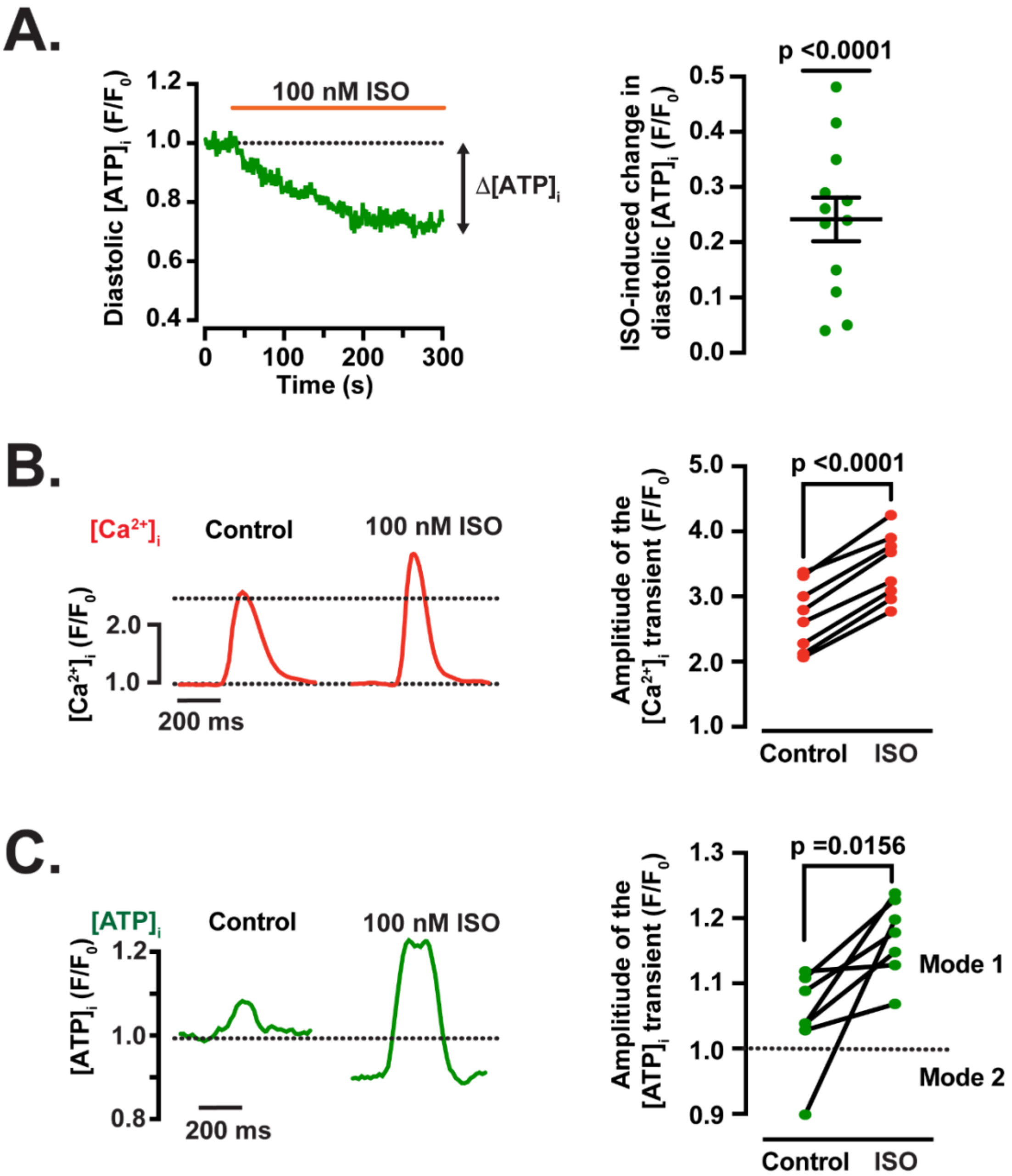

SI appendix Figure S9. Activation of β-adrenergic signaling decreases diastolic and increases systolic $[ATP]_i$. (A) Time course of $[ATP]_i$ in a quiescent ventricular myocyte before and after the application of the β-adrenergic agonist isoproterenol (ISO; 100 nM). The double headed arrow indicates the change in $[ATP]_i$ in response to ISO. The scatter plot shows the change in diastolic $[ATP]_i$ from multiple experiments (N = 3, n = 7). Action potential-evoked global $[Ca^{2+}]_i$ **(B)** and $[ATP]_i$ **(C)** transients from representative cells under control conditions and after ISO-treatment. The plots next to each of these traces summarize the results from multiple experiments and show the change in the amplitude of $[Ca^{2+}]_i$ and $[ATP]_i$ transients in the same cells before and after ISO (N = 3, n = 8). All significant values are provided from a paired *t*-test.

References

1. M. A. Lobas *et al.*, A genetically encoded single-wavelength sensor for imaging cytosolic and cell surface ATP. *Nature Communications* **10**, 711 (2019).
2. T. Shioya, A Simple Technique for Isolating Healthy Heart Cells from Mouse Models. *The Journal of Physiological Sciences* **57**, 327-335 (2007).
3. A. M. Krstic, A. S. Power, M. L. Ward, Visualization of Dynamic Mitochondrial Calcium Fluxes in Isolated Cardiomyocytes. *Front Physiol* **12**, 808798 (2021).
4. M. Maravall, Z. F. Mainen, B. L. Sabatini, K. Svoboda, Estimating intracellular calcium concentrations and buffering without wavelength ratioing. *Biophys J* **78**, 2655-2667 (2000).
5. H. Cheng, W. J. Lederer, M. B. Cannell, Calcium sparks: elementary events underlying excitation-contraction coupling in heart muscle. *Science* **262**, 740-744 (1993).
6. C. Du, G. A. MacGowan, D. L. Farkas, A. P. Koretsky, Calibration of the calcium dissociation constant of Rhod(2)in the perfused mouse heart using manganese quenching. *Cell Calcium* **29**, 217-227 (2001).
7. B. M. Drum, R. E. Dixon, C. Yuan, E. P. Cheng, L. F. Santana, Cellular mechanisms of ventricular arrhythmias in a mouse model of Timothy syndrome (long QT syndrome 8). *J Mol Cell Cardiol* **66**, 63-71 (2014).
8. D. Axelrod, D. E. Koppel, J. Schlessinger, E. Elson, W. W. Webb, Mobility measurement by analysis of fluorescence photobleaching recovery kinetics. *Biophys J* **16**, 1055-1069 (1976).
